# IL1β Expression Driven by Androgen Receptor Absence or Inactivation Promotes Prostate Cancer Bone Metastasis

**DOI:** 10.1158/2767-9764.CRC-22-0262

**Published:** 2022-12-02

**Authors:** Anthony DiNatale, Asurayya Worrede, Waleed Iqbal, Michael Marchioli, Allison Toth, Martin Sjöström, Xiaolin Zhu, Eva Corey, Felix Y. Feng, Wanding Zhou, Alessandro Fatatis

**Affiliations:** 1 Department of Pharmacology and Physiology, Drexel University College of Medicine, Philadelphia, Pennsylvania.; 2 Janssen Oncology, Spring House, Pennsylvania.; 3 AstraZeneca, Baltimore, Maryland.; 4 Department of Pathology and Laboratory Medicine, Perelman School of Medicine, University of Pennsylvania, Philadelphia, Pennsylvania.; 5 Department of Radiation Oncology, UCSF, San Francisco, California.; 6 Department of Urology, University of Washington, Seattle, Washington.; 7 Program in Translational and Cellular Oncology, Sidney Kimmel Cancer Center at Thomas Jefferson University, Philadelphia, Pennsylvania.

## Abstract

We report the inverse association between the expression of androgen receptor (AR) and IL1β in a cohort of patients with metastatic castration-resistant prostate cancer. We also discovered that AR represses the IL1β gene by binding an androgen response element half-site located within the promoter, which explains the IL1β expression in AR-negative (AR_NEG_) cancer cells. Consistently, androgen depletion or AR-pathway inhibitors (ARI) derepressed IL1β in AR-positive cancer cells, both *in vitro* and *in vivo*. The AR transcriptional repression is sustained by histone deacetylation at the H3K27 mark in the IL1β promoter. Notably, patients’ data suggest that DNA methylation prevents IL1β expression, even if the AR-signaling axis is inactive. Our previous studies show that secreted IL1β supports metastatic progression in mice by altering the transcriptome of tumor-associated bone stroma. Thus, in patients with prostate cancer harboring AR_NEG_ tumor cells or treated with androgen-deprivation therapy/ARIs, and with the IL1β gene unmethylated, IL1β could condition the metastatic microenvironment to sustain disease progression.

**Significance:**

IL1β plays a crucial role in promoting skeletal metastasis. The current standard of care for patients with prostate cancer inhibits the AR-signaling axis in tumor cells and will consequently unleash IL1β production. Thus, hormonal deprivation and AR inhibitors should be combined with targeting IL1β signaling, and screening for DNA methylation on the IL1β locus will identify patients that benefit the most from this approach.

## Introduction

At diagnosis, prostate cancer is driven by the androgen receptor (AR). Following local modalities such as radical prostatectomy or radiotherapy, patients presenting with biochemical recurrence are treated with androgen-deprivation therapy (ADT) and the majority show a favorable clinical response. However, 10%–20% of these patients will eventually develop castration-resistant prostate cancer (CRPC) within 5 years (1) and most of them present with metastatic lesions at diagnosis or within 3 years (2). At the metastatic stage (mCRPC), the molecular landscape is rewired for AR independence, when cancer cells reduce AR activity and often decrease or turn off AR expression (3). In line with this concept, we previously reported that skeletal metastases from 10 different patients with prostate cancer harbored substantial fractions of AR-negative (AR_NEG_) prostate cancer cells and confirmed these findings at the transcriptional level (4). Notably, the approval of two potent AR-pathway inhibitors (ARI), enzalutamide and abiraterone, has led to an increase in patients with mCRPC presenting with contingents of AR_NEG_ cancer cells (5). AR_NEG_ cancer cells are intrinsically resistant to ADT and ARIs and are intermixed with AR-positive (AR_POS_) cancer cells in metastatic lesions. Thus, lesions are heterogenous for AR-signaling status and response to the standard of care. This heterogeneity is compounded by the existence of distinct molecular phenotypes. AR_NEG_ cells can be classified as either neuroendocrine (NE), small-cell features (SCNPC), or double negative (DNPC) that lack both NE markers and AR (6), whereas AR_POS_ cancer cells can show either high (ARPC) or low AR expression (ARLPC). These observations bear high significance considering the functional cooperativity that exists among heterogenous tumor populations, particularly at metastatic sites (4, 7–10). To this point, DNPC phenotypes circumvent AR dependence by relying on alternate signaling pathways (5) and could also support the growth of ARPC cells under treatment with ADT or ARIs via unidentified mechanisms.

We have previously shown that the PC3-ML human prostate cancer cell line, which is AR_NEG_ (11, 12), expresses high levels of IL1β; in contrast, all AR_POS_ prostate cancer cell lines we tested uniformly lacked IL1β expression, in line with the inverse AR/IL1β correlation we observed in a small cohort of 10 patients (4).

Our interest in the potential role played by IL1β in mCRPC stems from our previous findings that the highly metastatic behavior of PC3-ML cells in preclinical models (13–17) could be dramatically mitigated by treatment with the IL1R antagonist anakinra (4). In addition, transgenic mice null for IL1R and grafted with PC3-ML cells developed significantly fewer and smaller skeletal lesions, suggesting IL1β secretion in the bone stroma forms a tumor-permissive niche (4). These findings were subsequently corroborated by others for bone metastatic prostate (18) and breast cancers (19–22).

Here we report a mechanism by which the AR directly represses IL1β expression involving histone deacetylation and a modulatory role of promoter and/or gene body methylation. These findings were validated in a larger cohort of patients with mCRPC showing high IL1β expression upon reduced AR activity, except when their IL1β locus was methylated. Thus, our study indicates that harboring AR_NEG_ cancer cells and/or being treated with ADT/ARIs increase tumor-derived IL1β which—upon its secretion—can instigate a stromal niche favorable to further tumor growth. This new evidence lends strong support to the utility of IL1β antagonism in prostate cancer, particularly for patients with an unmethylated IL1β promoter and/or gene.

## Materials and Methods

### Cell Lines and Cell Culture

Human LNCaP (RRID:CVCL_0395), DU-145 (RRID:CVCL_0105), and C4-2B (RRID:CVCL_4784) prostate cancer cells were purchased from ATCC. The human PC3-ML (RRID:CVCL_6E90) prostate cancer cell line was derived from the parental PC3 cell line as described previously (12). The PC3-ML and DU-145 cell lines were cultured in DMEM (Invitrogen) and the LNCaP and C4-2B cell lines were cultured in RPMI (Invitrogen)—both supplemented with 10% FBS (HyClone) and 0.1% gentamicin (Gibco). All cell lines were cultured at 37°C in a humidified incubator with 5% CO_2_. Each cell line was expanded to produce frozen aliquots and following resuscitation, were used for no more than 10 passages and no longer than 2 months. Cell line authentication was performed by IDEXX BioResearch using a single tandem repeat and conducted to determine the species of origin and rule out interspecies contamination by performing the *CellCheck 9 Plus* test. All cell lines were also tested for *Mycoplasma* contamination by IDEXX on a regular basis using PCR detection and only negative cells were used for this study. For *in vivo* experiments, LNCaP cells were transduced with lentiviral particles to express GFP and Luc2 Luciferase, and stable cell lines were produced through selection with the appropriate antibiotics for 3 weeks.

### Lentiviral Particle Production and Lentiviral Transduction

To achieve lentiviral transduction, HEK293T (RRID:CVCL_0063) cells were plated to be 90% confluent at the time of transfection. A total of 24 hours after plating, HEK293T cells were transfected with lentiviral envelope (pMD2.G, RRID:Addgene_12259) and packaging (pCMVR8.74, RRID:Addgene_22036) using lipofectamine 2000 (Thermo Fisher Scientific) and Opti-MEM–reduced serum medium (Thermo Fisher Scientific). Following overnight transfection, the transfection media was changed to DMEM supplemented with 10% FBS. Lentiviral particles were harvested at 24 and 48 hours posttransfection, and individual aliquots were stored at −80°C following passage through a 0.45 μm filter. For transduction, cell lines were incubated for 24 hours with lentivirus and 8 μg/mL polybrene (Santa Cruz Biotechnology).

### RNA-sequencing and Methylation Sequencing Data Analysis

RNA-sequencing (RNA-seq) and whole-genome bisulfite sequencing (WGBS) data from 100 mCRPC biopsies were acquired through a prospective multi-institution Institutional Review Board (IRB)-approved study (NCT02432001) conducted in accordance with the Declaration of Helsinki. Data processed as described previously (PMID: 30033370 and PMID: 32661416) and based on alignments to GRCh38.12. RNA-seq were normalized to transcripts per million (TPM) for further analysis. The TPM expression of genes was further normalized using z-score normalization (sample gene expression—mean gene expression of all samples/SD). To calculate the AR activity, we averaged the z-scores for the expression of nine AR-regulated genes that have previously been used to calculate an AR-activity score (PMID: 31515456, PMID: 29017058). The WGBS data were used to determine differential methylation patterns in the 100 mCRPC samples corresponding to the IL1β gene (ENSG00000125538.11, GENCODE v.28), which is annotated as having gene body from chr2:112829751 (transcription stop) to 112836903 (transcription start), with a negative strand orientation (PMID: 32661416); the promoter region of the gene was annotated as chr2:112836903 (transcription start) to 112838403 (1,500 bp upstream of transcription start). Differences in methylations and gene expressions between groups, segregated on the basis of high or low IL1β expression and or AR activity, were shown using the Wilcoxon rank-sum test. To identify IL1β methylation difference based on AR activity, we also analyzed methylation data for both cell lines. The analysis in cell lines was based on data obtained using the Infinium HM450 arrays (GSE491430) and methylations at specific CpG probes corresponding to the IL1β promoter were compared between DU-145 and PC3 cell lines.

### Patient-derived Samples and IHC Staining

Deidentified paraffin-embedded sections from patients with primary or metastatic prostate cancer were obtained from the Sidney Kimmel Cancer Center Biorepository of Thomas Jefferson University, a College of American Pathologists–accredited biorepository (accreditation #8427654), with support from the Cancer Center Grant 5P30CA056036-21. Acquisition of the biospecimens was approved through Thomas Jefferson University (Philadelphia, PA) under IRB #16P.726 upon obtaining written informed consent from patients. Paraffin-embedded human tissue sections were deparaffinized through a serial rehydration process including xylene, varying ethanol concentrations (100%, 95%, 90%, 70%), and deionized water. Rehydrated tissue sample underwent heat-induced antigen retrieval in a citrate buffer (pH 6.0), followed by a blocking step with 10% goat serum and 1% BSA. Primary antibodies for ACSL3 (PA5-42883, Invitrogen, RRID:AB_2609901) used at 1:200 and IL1β (ab9722, Abcam, RRID:AB_308765) used at 1:500 were applied to the sections and incubated overnight at 4°C. The sections were washed in 0.025% triton/TBS then placed in a methanol/hydrogen peroxide/TBS solution for endogenous peroxidase inhibition. An horseradish peroxidase (HRP)-conjugated secondary antibody (115-035-045, Jackson Immunoresearch) was used at 1:500 in combination with a 3,3′-Diaminobenzidine (DAB) chromogenic system. Hematoxylin was used as nuclear counterstain. The sections were washed in deionized water, dehydrated through varying ethanol concentrations, and immersed in xylene prior of being mounted with a coverslip using Permount (Thermo Fisher Scientific).

High-resolution digital images of stained tissue sections were acquired with a Hamamatsu S210 scanner and analyzed using QuPath software (v.0.2.3, RRID:SCR_018257), developed at the University of Edinburgh (Edinburgh, Scotland; ref. 23). Staining intensity was established by measuring average optical density of five randomly selected fields for each digital image with QuPath. GraphPad Prism 9.0 (RRID:SCR_002798) was used to generate graphs and for statistical analyses.

### Intratibial Grafting of Cancer Cells

Five-week-old male SCID mice (CB17-SCRF; Charles River) were housed in a germ-free barrier. At 6–8 weeks of age, mice were anesthetized using 80 mg/kg ketamine and 10 mg/kg xylazine. A total of 1 × 10^6^ LNCaP cells resuspended in DMEM/F12 media were delivered as a 50 μL suspension by bending the knee joint and carefully penetrating the articular surface of the tibia with an insulin syringe mounting a 30-gauge needle. Mice were sacrificed at specified timepoints following inoculation and tissues were prepared as described previously. All experiments were conducted in accordance with NIH guidelines for the humane use of animals. All protocols involving the use of animals were approved by the Drexel University College of Medicine Committee for the Use and Care of Animals.

### *In Vivo* Enzalutamide Treatment

Intratibial LNCaP tumors were allowed to establish and grow for 8 weeks. Animals were then randomly assigned to cages containing either control rodent diet (PicoLab Rodent Diet 20, catalog no. 5053, Test Diet) or the same rodent diet supplemented with 430 mg/kg enzalutamide (Med Chem Express, catalog no. HY-70002) for 14 days. The nutritional profiles for control and enzalutamide diets were equivalent with the exception of a green coloring agent in the enzalutamide diet to validate consumption after sacrifice.

### Tissue Preparation for FACS

Following animal sacrifice, tibiae were immediately harvested and freed of the surrounding soft tissues. A single-cell suspension was generated in 800 μL of DMEM/F12 by making repeated fine cuts with a razor blade in a sterile petri dish until no visible bone fragments remained. Prior to FACS sample acquisition, the cell suspension was centrifuged for 3 seconds to pellet any residual tissue fragments, and the supernatant immediately placed on ice.

### FACS of Cancer Cells Collected from Intratibial Tumors

FACS was conducted using a SH800 Cell Sorter (Sony Biotechnology). For all experiments, we used a 130 μm microfluidic sorting chip. Prior to sample acquisition, single-cell suspensions were obtained by filtration through a 70 μm cell strainer (Bel-Art SP Scienceware Flowmi). Sorting event rate was maintained below 2,000 events/second with a sample pressure of 3 or lower. GFP-positive tumor cell gating strategy was developed by flowing *in vitro* cultured GFP-positive tumor cells as well as cell suspensions obtained from knee joints of animals not inoculated with tumor cells and used either untouched or spiked with GFP-expressing tumor cells. The latter were sorted from the bone marrow suspension directly into buffer RLT.

### Gene Expression Analysis by qRT-PCR

Total RNA was isolated using the RNeasy Mini Kit (Qiagen) and stored at −80°C until qRT-PCR was performed. One-step qRT-PCR was performed using the TaqMan RNA-to-Ct 1-step kit (Thermo Fisher Scientific). TaqMan gene-specific primer and probe sets (FAM-MGB, Thermo Fisher Scientific) were used for GAPDH (Hs99999905_m1), IL1β (Hs01555410_m1), PMEPA1 (Hs00375306_m1), KLK3 (Hs02576345_m1), and AR (Hs00171172_m1). Results were analyzed using the Cloud Relative Quantification suite (Thermo Fisher Scientific). qRT-PCR was conducted using the QuantStudio 7 Flex Real-Time PCR system (Thermo Fisher Scientific). Fold change in gene expression was determined using the delta-delta *C*_t_ method with GAPDH as the internal reference gene.

### *In Vitro* Androgen Deprivation and Enzalutamide Treatment of Human Prostate Cancer Cells

For all experiments, LNCaP or C4-2B cells were plated 24 hours prior to treatment to be 70% confluent at the time of treatment. For the enzalutamide dose-escalation study, cells were treated for 48 hours with the indicated concentrations of drug (Med Chem Express, catalog no. HY-70002). For the time-course experiments, cells were treated with 1 μmol/L enzalutamide for the indicated duration. When necessary, enzalutamide-containing media was replenished every 3 days. For the enzalutamide-removal experiment, LNCaP cells were treated for 10 days with 1 μmol/L enzalutamide. After collecting 10-day treatment samples for transcript analysis, cells were washed twice with PBS and cultured under control conditions, with samples being collected every 2 days for 8 days for transcript analysis. For androgen-deprivation experiments, cells were cultured in media supplemented with charcoal-stripped serum (CSS, Sigma-Aldrich), for the indicated time period. At all experimental endpoints, cells were lysed for either protein or RNA extraction.

### SDS-PAGE and Western Blotting

Cell lysates were collected with RIPA lysis and extraction buffer (#89900 Thermo Fisher Scientific) containing a phosphatase inhibitor cocktail (Calbiochem), a protease inhibitor cocktail (Calbiochem), 10% glycerol, and 0.5 mol/L Ethylendiaminetetraacetic acid (EDTA) (Thermo Fisher Scientific). A BCA protein assay (Pierce) was used to determine protein concentrations and 50 μg of proteins were loaded on 10% polyacrylamide gels and then transferred onto Immobilon polyvinylidene difluoride membranes (Millipore Corporation). Membranes were blocked for 1 hour at room temperature with 0.1% Tween-20/TBS with 5% (w/v) powdered milk. AR was detected using a primary antibody (Abcam, ab108341, RRID:AB_10865716) used at 1:2,000, diluted in 0.1% Tween-20, 5% dry milk in TBS and incubated overnight at 4°C. GAPDH (Cell Signaling Technology, 5174S, RRID:AB_10622025) was used at 1:5,000, in 0.1% Tween-20, 5% TBS and incubated overnight at 4°C. A secondary, HRP-conjugated antibody (Pierce) was used at 10 ng/mL. Blotted membranes were processed with SuperSignal Femto chemiluminescence substrates (Pierce) and visualized using a FluorChem imaging system (ProteinSimple).

### Generation of AR-expressing Constructs and Lentiviral Particle Transduction

The human AR gene was amplified from pCMV-hAR (RRID:Addgene_89078) by PCR for Gibson Assembly (New England BioLabs). The pHAGE TRE dCas9-KRAB plasmid (RRID:Addgene_50917) was modified to replace G418 resistance with hygromycin resistance. dCas9 was replaced by the AR, putting it under control of the tetracycline response element. PCR was used to amplify the cytomegalovirus (CMV) promoter and AR gene from pCMV-hAR (Addgene), which was then inserted into the pLenti CMV/TO Hygro empty construct (RRID:Addgene_17484) using Gibson Assembly. Following lentiviral transduction of PC3-ML wild-type cells, inducible and stable AR expression was confirmed by qRT-PCR and Western blotting. All experiments were initiated 72 hours posttransduction. When indicated, transduced cells were treated with 10 nmol/L dihydrotestosterone (DHT) for 24 hours.

### Quantification of IL1β Protein by ELISA

To quantify intracellular IL1β protein, cell lysates were collected with RIPA lysis and extraction buffer (Thermo Fisher Scientific) containing a phosphatase inhibitor cocktail (Calbiochem), protease inhibitor cocktail (Calbiochem), 10% glycerol, and 0.5 mol/L EDTA (Thermo Fisher Scientific). All cell lysate samples were collected at a volume no greater than 40 μL and stored at −20°C until analysis. The protein concentration of each sample was determined using a BCA assay immediately prior to ELISA analysis, and cell lysates were brought to a total volume of 200 μL using RIPA buffer (Thermo Fisher Scientific). To quantify secreted IL1β protein, cell culture supernatant was collected at completion of the experiment. Supernatants were centrifuged at 500 × *g* for 5 minutes at 4°C, then transferred to new collection tubes and stored at −80°C until analysis. Samples were loaded, in technical duplicates, on precoated 96-well plates of the Human IL1β/IL1F2 Quantikine ELISA kit (R&D Systems). Assay was completed in accordance with manufacturer protocol. Results of assay were analyzed with ElisaAnalysis.com software v3.2 (Leading Technology Group). Assay readouts were reported as results normalized to starting protein concentration for each sample.

### Chromatin Immunoprecipitation Quantitative PCR

Chromatin immunoprecipitation (ChIP) was performed using the ChIP-IT Express Chromatin Immunoprecipitation Kit (ActiveMotif). A ChIP-validated human AR antibody (6 μg, ActiveMotif 39781, RRID:AB_2793341) was bound to 25 μL of magnetic Dynabeads (Thermo Fisher Scientific). ChIP was simultaneously performed with beads only to determine background. LNCaP cells were cultured in a 150 mm culture dish under normal serum-containing conditions. At 80% confluency, culture medium was replaced with a medium containing CSS for 48 hours. Cells were then treated with 10 nmol/L DHT for 3 hours with or without 1 μmol/L enzalutamide. Proteins were cross-linked to DNA with 1% formaldehyde for 10 minutes, followed by quenching with glycine. Chromatin was fragmented by 30 on-off cycles of sonication. Samples were purified with the ChIP DNA purification kit (ActiveMotif). A primer pair for qPCR was designed using Primer3web to span the IL1β promoter ARE with an amplicon size of 133 bp. Primers were checked for specificity using BLAST. Optimal Tm was set to 59°C with a difference no greater than 1°C. qPCR was performed using the PowerUp SYBR Green Master Mix (Thermo Fisher Scientific). The ChIP-IT Control Kit (ActiveMotif) was used to confirm a successful immunoprecipitation (IP) and to determine background. A primer set for KLK3 (ActiveMotif) was used to demonstrate successful IP of AR-bound chromatin. AR enrichment was calculated using percent input method and presented as fold change. This protocol was followed to determine H3K27Ac enrichment in the IL1β promoter using a ChIP-validated histone H3K27ac antibody (10 μg, ActiveMotif 39034, RRID:AB_2561016) following 48 hours of culture under androgen-deprived conditions, relative to culture under androgen-containing conditions. A primer set for PABPC1 (ActiveMotif) was used to demonstrate successful IP of H3K27Ac-bound chromatin.

### ChIP Primer Sequences

See Supplementary Table S1.

### IL1β Promoter-luciferase Reporter Constructs

An IL1β promoter-luciferase fusion construct (SwitchGear Genomics) was transferred to the pGFP-c-shLenti vector (Origene) using the NEBuilder HiFi DNA assembly master mix (NEB), allowing for Lentiviral transduction and TurboGFP expression for normalization. A small fragment of the IL1β promoter containing the ARE half-site was deleted using an additional primer pair during transfer to the pGFP-c-ShLenti vector through a three-part Gibson assembly reaction. Cloning was performed with competent Stbl3 E. Coli. The LightSwitch Luciferase Assay Reagent (SwitchGear Genomics) was used for quantification of luciferase expression.

### Trichostatin A Treatments

LNCaP and PC3-ML cells were plated 24 hours prior to treatment and—when 70% confluent—were treated for 24 hours with 400 nmol/L trichostatin A (TSA; Sigma-Aldrich). RNA was then isolated for downstream transcript analysis by qRT-PCR.

### *In Vitro* Treatment with BET Inhibitors

LNCaP and PC3-ML cells were plated 24 hours prior to treatment and—when 70% confluent—were treated for 24 hours with the indicated concentrations of the BET inhibitors JQ1 and PLX51107 (a gift from Dr. Edward Hartsough). In the indicated experiments, LNCaP cells were also treated with 1 μmol/L enzalutamide. RNA was then isolated for downstream transcript analysis by qRT-PCR.

### *In Vitro* Treatment with 5-azacytidine

PC3-ML and DU-145 cells were plated 24 hours prior to treatment and—when 25% confluent—were treated for 72 hours with 5 μmol/L 5-Azacytidine. RNA was then isolated for downstream transcript analysis by qRT-PCR.

### Statistical Analyses

Results are reported as mean ± SEM. When comparing two experimental groups, statistical significance was determined by Student *t* test with Welch correction (GraphPad Prism 5.0). When comparing multiple experimental groups, statistical significance was determined by one-way ANOVA with Dunnett post-test. In both cases, statistical significance was achieved by *P* < 0.05.

## Results

### Repression of AR-activated Genes Correlates with High Expression of IL1β in Patients with mCRPC

**FIGURE 1:**
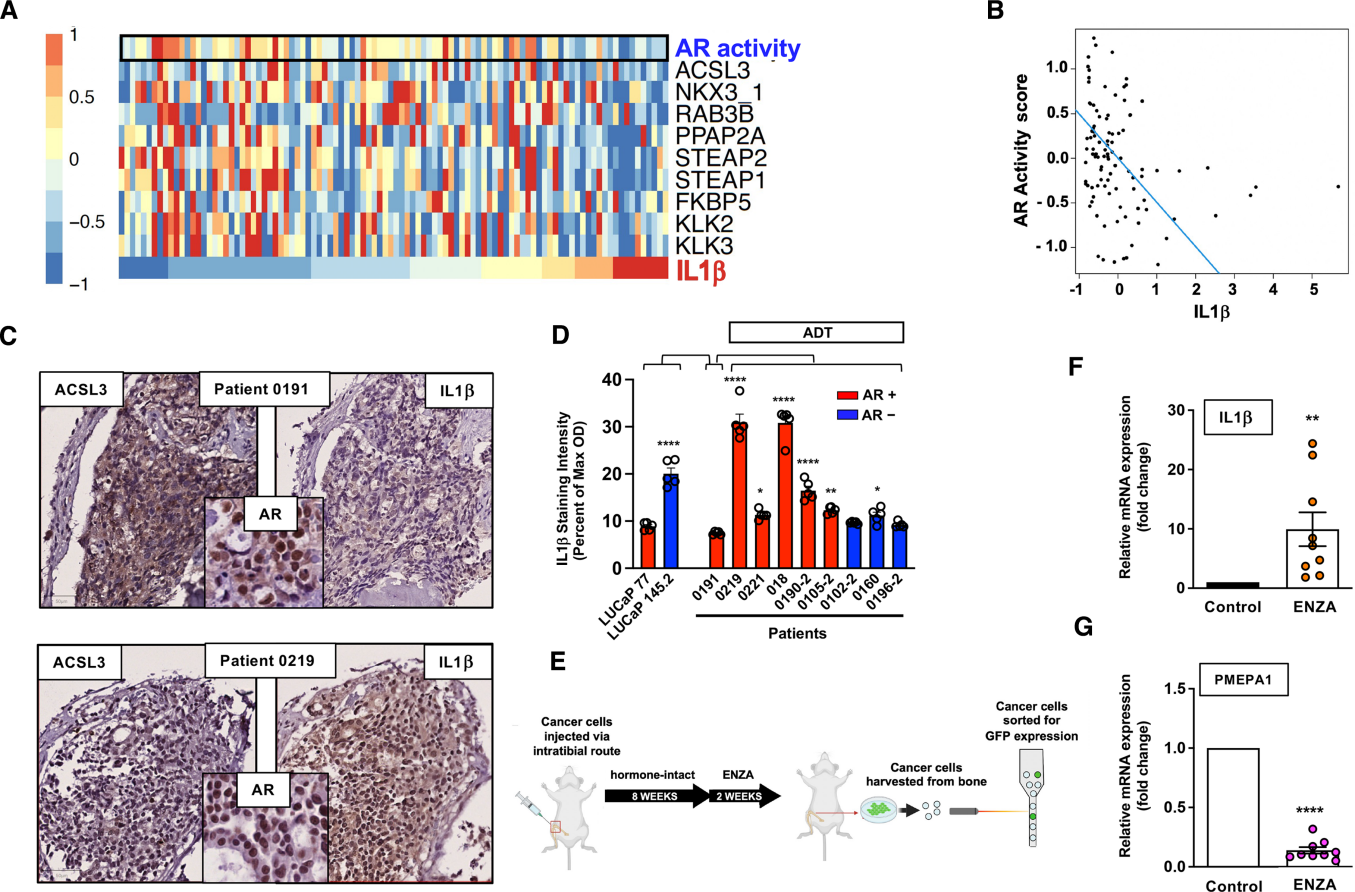
IL1β expression inversely correlates with an active AR-signaling axis in patients with prostate cancer and in an animal model of bone metastatic prostate cancer. **A,** The global gene expression profiles of 100 metastases from patients with CRPC were analyzed for the expression of IL1β and nine established AR-regulated genes. The results are presented as a heatmap showing z-score normalized IL1β expression levels in relation to an AR-activity index, obtained by averaging the z-score normalized expression levels of all nine AR-regulated genes. Samples are ordered from lowest to highest IL1β expression from left to right in the heatmap. **B,** Within the set of CRPC metastases, IL1β expression inversely correlates with the AR-activity index. Correlation of AR activity averaged z-score versus z-score normalized IL1β, for the 100 CRPC samples, is shown in the scatterplot. **C,** Representative images of IHC detection of IL1β, AR and AR-regulated gene ACSL3 performed on consecutive tissue sections from patients with mCRPC. **D,** IHC results quantified in five different fields from digital images obtained for each patient with mCRPC and compared with similar analysis conducted on two prostate PDXs with different AR expression and activity status. **E,** Experimental schematic of the intratibial tumor models used to assess the effects of AR inactivation by enzalutamide on IL1β expression. **F,** Increase in IL1β transcript levels detected in tibial tumor bearing mice treated with enzalutamide (ENZA) as compared with control animals. **G,** Validation of the *in vivo* enzalutamide pharmacologic effect on AR shown by the mitigation of the expression of the AR-regulated gene PMEPA1. (**B,** Spearman coefficient: −0.352; **E,** *, *P* = 0.0174; **, *P* = 0.017; ****, *P* < 0.0001; **F,** **, *P* = 0.0035; all data points were compared with patient 0191, which is treatment naïve; **G,** ****, *P* < 0.0001). Data are presented as mean values ± SEM. One-way ANOVA or Student *t* test.

Using the Oncomine database (24), we have previously shown that IL1β expression is increased in tumor tissue as compared with normal prostate tissue and that AR_NEG_ cells in patients with mCRPC express high levels of IL1β while AR_POS_ cells lack IL1β expression (4, 14). Here we sought to expand these earlier findings by correlating AR transcriptional activity with relative IL1β expression. To this end, we analyzed global gene expression data of fresh-frozen core biopsies of metastases collected from 100 patients with CRPC (25). To identify samples with impaired AR-signaling axis, from patients either exposed to ADT/ARIs or with metastases harboring AR_NEG_ cancer cells, we examined nine AR-regulated genes (KLK3, KLK2, FKBP5, STEAP1, STEAP2, PPAP2A, RAB3B, ACSL3, and NKX3-1; refs. 26, 27). By averaging the z-score normalized expression levels of all nine AR-regulated genes, we generated an AR-activity index and used it to measure AR activity in 100 CRPC cases with matched gene expression and DNA methylation data: this revealed a strong inverse correlation between AR transcriptional activity and IL1β expression (Fig. 1A and B; Table 1). These results were corroborated by assessing AR/IL1β protein expression by IHC in two prostate patient-derived xenografts (PDX), LUCaP 145.2 (AR_NEG_) and LUCaP 77 (AR_POS_; ref. 28), and nine bone specimens from patients with metastatic prostate cancer either treatment-naïve (patient 0191) or exposed to ADT/ARIs. We found that the absence of AR expression (LUCaP 145.2 and patients 0102-2, 0160, and 196-2) or suppression of AR activity by ADT/ARIs—assessed by reduced or absent ACSL3 expression—were accompanied by an increase in IL1β expression (Fig. 1C and D).

**TABLE 1:** Correlation between the z-score normalized IL1β expression and nine AR-regulated genes

AR-regulated genes	Correlation with IL1β expression	Wilcoxon test
**KLK3**	**−0.233822188**	**0.023985358**
**KLK2**	**−0.222084874**	**0.047470798**
**FKBP5**	**−0.10107532**	**0.457313212**
**STEAP1**	**−0.338317892**	**0.005129863**
**STEAP2**	**−0.414226393**	**0.001278009**
**PPAP2A**	**−0.300453651**	**0.009158984**
**RAB3B**	**−0.019562191**	**0.047470798**
**NKX3_1**	**−0.20384283**	**0.061212118**
**ACSL3**	**−0.132998896**	**0.044473089**
**AR activity**	**−0.352107436**	**0.000456029**

NOTE: The Wilcoxon test results compare gene expression differences in AR-regulated genes for high (z-score > = 0.5, *n* = 17) and low (z-score < 0.5, *n* = 83) IL1β-expressing CRPC samples.

### Androgen deprivation and ARI Treatment Both Induce IL1β Expression *In Vivo*

More than 80% of patients with CRPC develop skeletal metastases (29, 30). Thus, to model the AR/IL1β inverse correlation from patients with skeletal metastatic disease, we grafted GFP-expressing LNCaP prostate cancer cells directly into the tibiae of hormone-intact mice. After 8 weeks, animals were fed with either a regular diet or an enzalutamide-supplemented diet for 2 weeks. Both sets of animals developed tumors, with enzalutamide-treated animals showing smaller tumors that nonetheless never regressed. Tumors from control and treated mice were then harvested to collect cancer cells separately from murine host cells by using FACS and gating for GFP (Fig. 1E). We found that LNCaP cells from enzalutamide-treated animals robustly upregulated IL1β transcript expression compared with cancer cells collected from mice on a control diet (Fig. 1F). The effective inactivation of the AR signaling axis by enzalutamide was confirmed by the strong reduction in transcript levels of the AR-regulated gene PMEPA1 (ref. 31; Fig. 1G).

### The AR Represses IL1β Transcription

**FIGURE 2:**
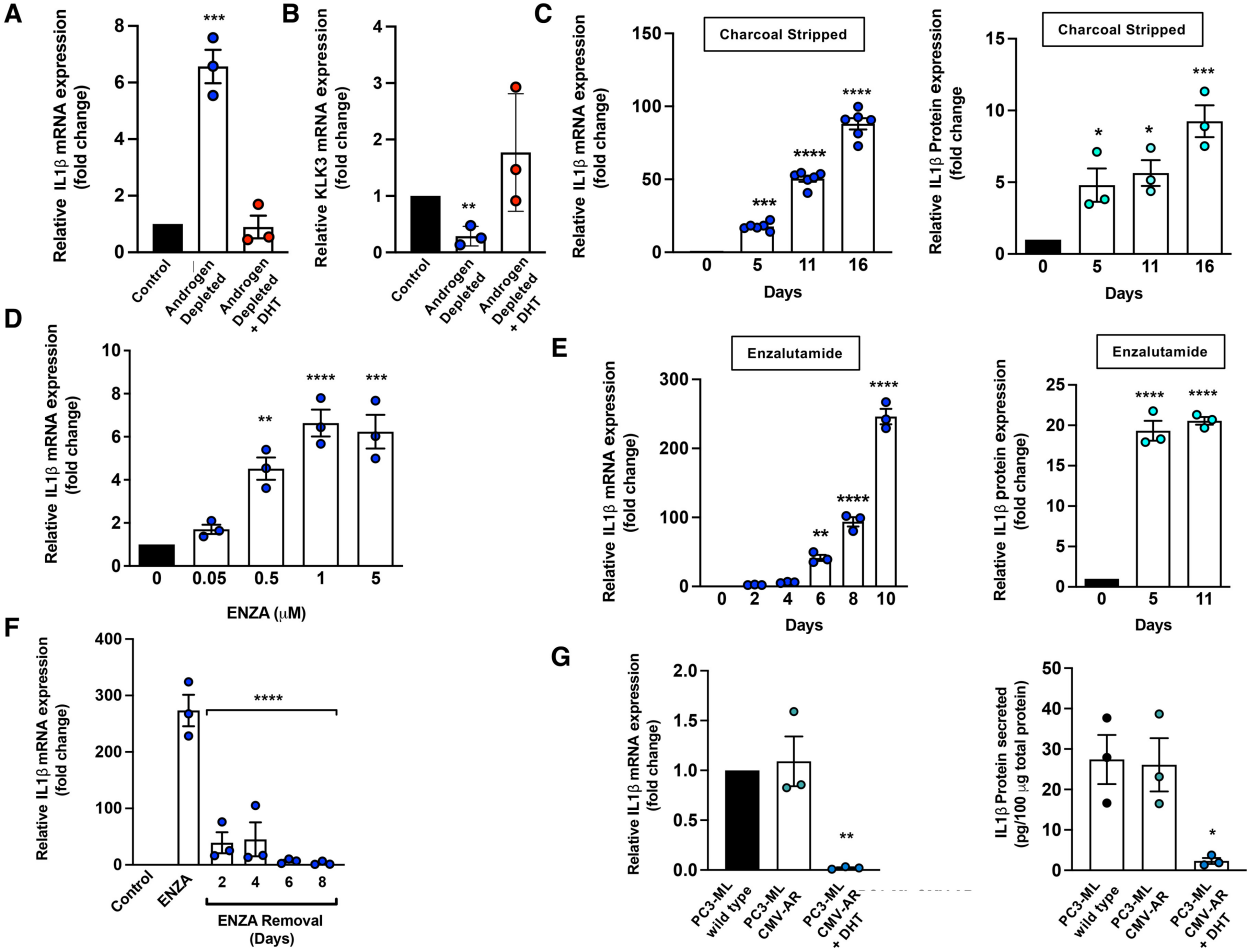
The AR represses IL1β expression at the transcriptional level *in vitro*. **A,** LNCaP cells cultured under androgen-depleted conditions for 24 hours expressed significantly higher IL1β transcript relative to those cultured under androgen-containing conditions. LNCaP cells cultured under androgen-depleted conditions supplemented with 10 nmol/L DHT showed no significant difference in IL1β expression relative to those cultured under androgen-containing conditions. **B,** Downregulation of the AR-regulated gene KLK3 in androgen-depleted conditions and restoration of its expression by 10 nmol/L DHT. **C,** LNCaP cells cultured under androgen-depleted conditions showed a significant time-dependent increase in IL1β transcript expression (left) and intracellular IL1β protein expression (right), relative to LNCaP cells cultured under androgen-containing conditions. Protein expression for each group was normalized to total protein concentration. **D,** LNCaP cells treated with enzalutamide (ENZA) for 48 hours demonstrated a dose-dependent increase in IL1β transcript expression relative to untreated control cells. **E,** LNCaP cells treated with 1 μmol/L enzalutamide showed a time-dependent increase in IL1β transcript expression (left) and intracellular IL1β protein expression (right), relative to untreated control cells. Protein expression for each group was normalized to total protein concentration. **F,** LNCaP cells treated with 1 μmol/L enzalutamide for 10 days significantly upregulated IL1β transcript expression, with removal of enzalutamide resulting in IL1β transcript levels progressively returning to that of untreated control cells. **G,** PC3-ML cells were transduced with a CMV-driven AR expression construct (PC3-ML CMV-AR) and 72 hours later were left untreated or exposed for 24 hours to 10 nmol/L DHT. The expression of IL1β transcript (left) and secreted IL1β protein (right) were significantly repressed in PC3-ML CMV-AR cells treated with DHT relative to control wild-type PC3-ML cells. [**A,** ***, *P* = 0.0001; **B,** **, *P* = 0.002; **C,** ***, *P* = 0.0001; ****, *P* < 0.0001 (left); *, *P* = 0.0478; *, *P* = 0.0187; ***, *P* = 0.0006 (right); **C,** **, *P* = 0.0022; ****, *P* < 0.0001; ***, *P* = 0.0001; **D,** ****, *P* < 0.0001; **E,** ****, *P* < 0.0001; **F,** **, *P* = 0.0054 (left); *, *P* = 0.0250 (right). Data are presented as mean values ± SEM. One-way ANOVA].

On the basis of the evidence that enzalutamide treatment upregulates IL1β in the skeletal tumors of mice, we next sought to tease out the mechanisms downstream of AR inactivation that derepress IL1β transcription. First, we cultured hormone-dependent LNCaP cells in androgen-depleted conditions and quantified the resulting IL1β transcript levels after 24 hours, observing a 6-fold upregulation as compared with the untreated controls (Fig. 2A). Consistently, when DHT was added to androgen-deprived culture medium, the IL1β transcript levels did not increase and remained similar to controls, indicating an inverse correlation between IL1β transcription and AR signaling (Fig. 2A). The effective repression of AR transcriptional activity in androgen-depleted conditions was confirmed by the downregulation of the AR-regulated gene KLK3, for which the expression was restored by DHT addition to the androgen-depleted medium (Fig. 2B). To determine whether the upregulation of IL1β was an acute or sustained response, we extended the duration of androgen depletion up to 16 days. In these conditions, IL1β expression increased over time and did not subside or plateau. After 16 days of androgen deprivation, LNCaP cells showed a 100-fold increase in IL1β transcript expression and a 10-fold increase in IL1β intracellular protein (Fig. 2C). Next, based on the previous results from *in vivo* experiments with intratibial tumors, we treated LNCaP cells with enzalutamide and observed a dose-dependent increase in IL1β transcript expression (Fig. 2D). As observed for androgen deprivation, extending enzalutamide treatment for 11 days increased both IL1β transcript expression and intracellular protein over time (Fig. 2E). To ascertain whether prolonged enzalutamide treatment drives a permanent phenotype alteration, as opposed to a transient transcriptional upregulation of the IL1β gene, we treated LNCaP cells with enzalutamide for 10 days, then removed the drug and quantified the IL1β transcript levels over the following 8 days (Fig. 2F). In these conditions, IL1β transcription progressively returned to the baseline control levels, indicating that the functional inactivation of AR increases IL1β expression by a reversible transcriptional modulation (Fig. 2F). We then aimed to determine whether growth under androgen-deprived conditions or enzalutamide treatment upregulates IL1β expression also in the androgen-independent and AR_POS_ C4-2B cells, a subline derived from LNCaP cells (32, 33). C4-2B cells exposed to enzalutamide similarly showed both a dose-dependent and time-dependent increase in IL1β transcript expression (Supplementary Fig. S1A and S1B). Consistently, removal of enzalutamide following long-term treatment of C4-2B cells returned IL1β expression to control levels (Supplementary Fig. S1C). The same cells kept in androgen-deprived conditions for 15 days significantly increased IL1β transcript levels, while restoring androgen-containing conditions returned IL1β expression to control levels after 4 days (Supplementary Fig. S1D).

Because targeting the AR-signaling axis upregulated IL1β in AR_POS_ cell lines, we conducted complementary experiments where we exogenously expressed AR in PC3-ML cells, a subline derived from the AR_NEG_, parental PC3 cells (12) that displays high IL1β levels and aggressive metastatic behavior in animal models (4). PC3-ML cells transduced with a stable AR overexpression construct (PC3-ML CMV-AR; Supplementary Fig. S2A) showed ablation of IL1β transcription and protein expression when cells were exposed to DHT (Fig. 2G). Similar results were obtained in PC3-ML cells transduced with a doxycycline-inducible AR overexpression construct (PC3-ML TRE-AR; Supplementary Figs. S2A, S2B, and S3). Taken together, these data suggest that an active AR-signaling axis directly represses IL1β expression, and that AR inhibition relieves the transcriptional repression of IL1β.

### The AR Represses IL1β Through Chromatin Binding

**FIGURE 3:**
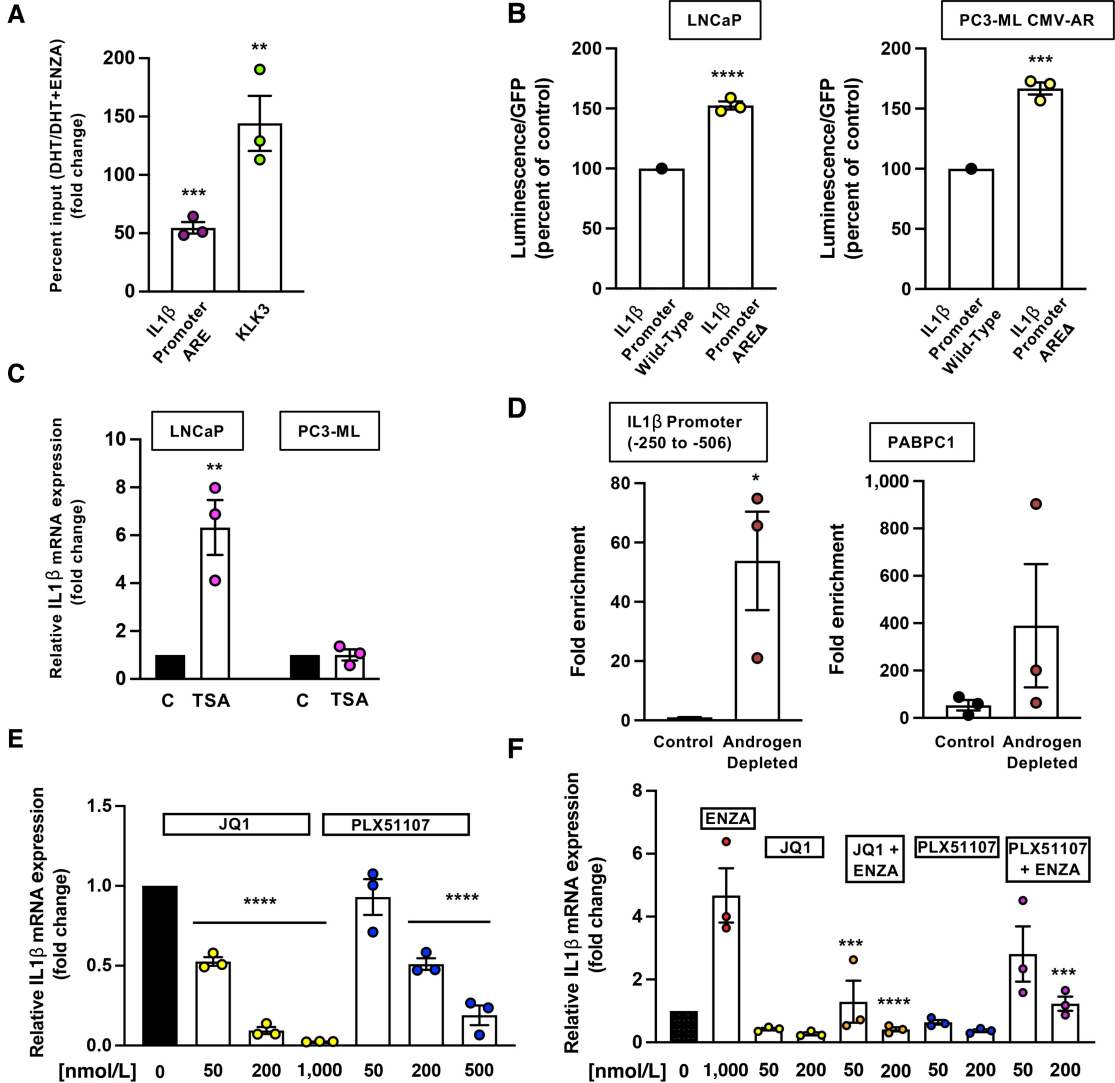
IL1β transcription is regulated by AR-chromatin binding and modulated by histone acetylation. **A,** ChIP-qPCR was used to demonstrate a significantly enriched AR-chromatin binding at the ARE half-site within the IL1β promoter (−576) with 10 nmol/L DHT in comparison with DHT and 1 μmol/L enzalutamide (ENZA) treatment. Prior to ChIP, LNCaP cells were cultured under androgen-deprived conditions for 48 hours followed by 3 hours of treatment with 10 nmol/L DHT with or without 1 μmol/L enzalutamide, which was added 30 minutes prior to DHT treatment. A known site of AR binding in an enhancer for KLK3 was used to demonstrate ChIP specificity for the AR. In both cases, significant AR-chromatin enrichment was observed in the presence of DHT as compared with DHT and enzalutamide treatment and expressed using the percent input method. **B,** IL1β promoter activity was quantified in LNCaP cells (left) and PC3-ML CMV-AR cells (right) using full-length and ARE-deleted (AREΔ) IL1β promoter-driven luciferase reporter constructs, with the AREΔ construct resulting in a significant increase in luminescence in both the LNCaP and PC3-ML CMV-AR cells when compared with the wild-type construct. PC3-ML cells transduced with the CMV-driven AR construct were treated with 10 nmol/L DHT. Mean luminescence values were normalized to GFP fluorescence intensity. **C,** LNCaP and PC3-ML cells treated with 400 nmol/L TSA for 24 hours resulted in LNCaP cells significantly upregulating IL1β transcript expression relative to untreated control cells, while PC3-ML cells treated with TSA showed no significant difference in IL1β transcript expression relative to the untreated control cells. **D,** ChIP-qPCR was used to demonstrate that 48 hours of culture under androgen-deprived conditions results in significant enrichment of the H3K27Ac histone modification, relative to androgen-containing conditions, within the IL1β promoter downstream of the ARE half-site and upstream to the transcription start site (left). Specificity of H3K27Ac ChIP-qPCR was validated using a known site of enrichment in PABPC1 (right). **E,** PC3-ML cells treated for 24 hours with the BET protein inhibitors JQ1 or PLX51107 demonstrated a significant dose-dependent downregulation of IL1β transcription relative to untreated control cells. **F,** LNCaP cells were treated for 24 hours with either 1 μmol/L ENZA, JQ1 or PLX51107, or a combination of JQ1 or PLX51107 and ENZA. ENZA treatment resulted in a significant upregulation in IL1β transcript expression relative to untreated cells, but combination treatment with JQ1 or PLX51107 inhibited the ENZA-mediated upregulation thus resulting in no significant difference in IL1β expression relative to the untreated control cells. [**A,** ***, *P* = 0.0004; **, *P* = 0.0037; **B,** ****, *P* < 0.0001 (left); ***, *P* = 0.0002 (right); **C,** **, *P* = 0.0098; **D,** *, *P* = 0.0337; **E,** ****, *P* < 0.0001; **F,** ***, *P* = 0.0001. Data are presented as mean values ± SEM. Student *t* test or one-way ANOVA].

Because the AR canonically exerts its activity through chromatin binding, we examined the IL1β locus for the presence of consensus androgen response elements (ARE). Conventional AREs are 15-bp palindromic sequence, but the AR can also bind to ARE half-sites with the sequence 5′-AGAACA-3′ (34). The intergenic region of the IL1β locus contains three ARE half-sites, whereas the IL1β promoter contains one ARE half-site, located 576 bp upstream of the transcription start site (35). While it has been widely reported that the AR can activate transcription by binding to distal enhancers, recent evidence has revealed that the AR can also repress transcription by binding the promoters of a subset of genes—including hTERT (36), MUC1 (37), and PEG10 (38). Thus, we determined whether the AR directly interacts with the IL1β promoter using ChIP and quantitative PCR (ChIP-qPCR). Interestingly, in LNCaP cells, we found that AR binding to chromatin is significantly enriched at the ARE half-site within the IL1β promoter and impaired by enzalutamide (Fig. 3A). We validated successful immunoprecipitation of AR-bound chromatin using an established AR–chromatin binding site within KLK3, an AR-regulated gene (ref. 39; Fig. 3A).

We next assessed the functionality of the AR–chromatin interaction at the ARE within the IL1β promoter by transducing LNCaP and PC3-ML cells with an IL1β promoter-driven luciferase reporter system. The promoter sequence consisted of 964 bp upstream of the wild-type IL1β transcription start site and we removed a small DNA sequence containing the ARE half-site located at −576 to specifically impair AR binding to the promoter (AREΔ IL1β promoter). When expressed In LNCaP cells, the AREΔ IL1β promoter showed significantly higher luciferase activity than its wild-type counterpart, indicating that the ARE half-site, at least in part, serves to repress IL1β transcription (Fig. 3B). In line with these findings, PC3-ML cells showed no difference in luciferase activity between the wild-type promoter and the AREΔ IL1β promoter (Supplementary Fig. S4), whereas the same cells transduced with a stable AR overexpression construct showed significantly higher luciferase activity from the AREΔ IL1β promoter than from its wild-type counterpart (Fig. 3B). Taken together, these results implicate an AR-chromatin interaction at the IL1β promoter ARE half-site as a main culprit for IL1β repression.

### IL1β Expression is Promoted by Histone Acetylation

To elucidate the mechanistic underpinning for how the AR-chromatin interaction represses IL1β transcription, we interrogated the ENCODE project (40). This revealed an enriched acetylation of the lysine at N-terminal position 27 of histone protein H3 (H3K27Ac) in the IL1β promoter near the ARE half-site. The H3K27Ac epigenetic mark has been characterized as an activator of transcription that is mainly found close to the TSS of several genes (41). We therefore hypothesized that the AR represses IL1β transcription by recruiting one or more histone deacetylases (HDAC)—enzymes that remove acetyl groups from histone proteins—in a similar fashion to AR-mediated repressive mechanisms reported by others for genes such as CDHE (42) and CCND1 (43). To test this hypothesis, we treated LNCaP and PC3-ML cells with the pan-HDAC inhibitor TSA and quantified the resulting IL1β transcript levels. TSA treatment significantly increased IL1β transcript levels in the LNCaP cells but not in the AR_NEG_ PC3-ML cells, thus ruling out AR-unrelated effects caused by HDAC inhibition. (Fig. 3C). These findings strongly implicate histone deacetylation in AR-mediated repression of IL1β transcription.

We next sought to determine whether androgen depleted conditions enrich the H3K27Ac modification at the IL1β promoter, which would permit IL1β transcription. Using ChIP-qPCR, we observed that the H3K27Ac modification is completely absent from the IL1β promoter in LNCaP cells cultured in androgen-containing conditions (Fig. 3D). In contrast, when the LNCaP cells were kept in androgen-depleted conditions, H3K27Ac was enriched at the IL1β promoter from the ARE half-site to the TSS (Fig. 3D). We validated the specificity of our ChIP-qPCR methodology by examining a known site of H3K27Ac enrichment for the PABPC1 promoter (44).

### IL1β Expression is Mediated by Bromodomain and Extraterminal Motif Proteins

Bromodomain and extraterminal motif (BET) proteins, which mediate the downstream effects of H3K27Ac chromatin modifications, are also known to promote gene expression. Specifically, bromodomain proteins such as BRD4 promote transcription by binding acetylated-histone protein H3 and recruiting positive transcription elongation factor (P-TEFb) to the promoter (45). To ascertain whether BET proteins promoted IL1β transcription, we used two different BET protein inhibitors (BETi), JQ1 and PLX51107. Both compounds are potent inhibitors of BRD4, but they also block the activity of other BET family members such as BRD2, BRD3, and BRDT (46, 47). Treatment of PC3-ML cells with either JQ1 or PLX51107 resulted in a dose-dependent inhibition of IL1β transcription (Fig. 3E). Consistently, both BETi compounds dose-dependently blocked the enzalutamide-induced upregulation of IL1β in the LNCaP cells (Fig. 3F).

### DNA Methylation Inhibits IL1β Expression

**FIGURE 4:**
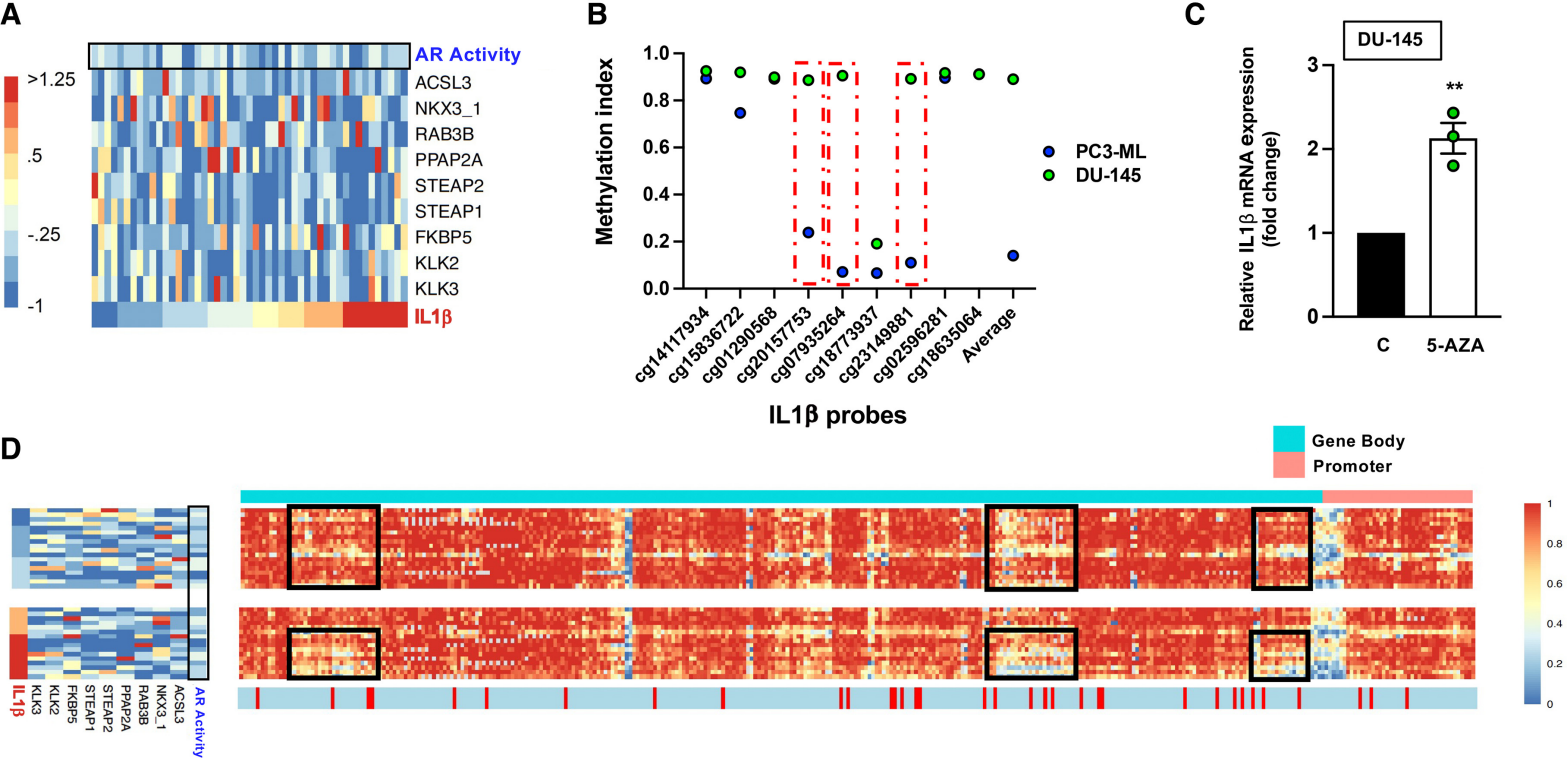
IL1β expression in prostate cancer is regulated, in part, by DNA methylation. **A,** Heatmap shows that subsets of patients with low AR activity gene expression (<0 z-score, *n* = 49) have varying degrees of high and low IL1β gene expression. Samples are ordered from lowest to highest IL1β expression from left to right in the heatmap. **B,** Three CpG sites on the IL1β promoter of DU-145 cells are hypermethylated in comparison with PC3 cells; For convenience, we also plot the average methylation difference across the three CpG probes between these two cell lines. **C,** Treatment of DU-145 cells with 5 μmol/L 5-azacytidine (5-Aza) for 72 hours resulted in a significant upregulation of IL1β transcript expression relative to untreated control cells. **D,** RNA-seq and WGBS data from the low AR CRPC subset (*n* = 49) were further subset based on having low IL1β expression (≤0.25 z-score, *n* = 18) or high IL1β expression (>0.5 z-score). Samples are ordered from highest to lowest IL1β expression from top to bottom in each respective heatmap and the ordering is matched between the methylation and gene expression heatmaps of both groups. Out of 346 CpG sites spanning IL1β promoter and gene body on chromosome 2, we found that 34 CpG sites (red marks) showed significant methylation in patients that failed to upregulate IL1β despite an inhibited AR-signaling axis. Black boxes indicate areas with the highest differences in methylation—including several CpG sites—among patients with opposite IL1β expression (**C**, **, *P* = 0.0035. Data are presented as mean values ± SEM. Student *t* test).

Although an inactive AR signaling allows the derepression of IL1β transcription, we found that this paradigm could not be uniformly confirmed in cell lines or patients’ samples. For instance, DU-145 prostate cancer cells are AR_NEG_ and yet fail to express the IL1β protein (4) or its transcript. Similarly, further analysis of the patient cohort previously used to define the inverse AR/IL1β association revealed that a fraction of the patients presenting with low AR activity unexpectedly lacked IL1β expression (Fig. 4A). On the basis of this evidence, we reasoned that additional mechanisms are likely to restrain IL1β expression when the AR is inactive. Previous studies, using whole-genome DNA methylation analysis and global transcription analysis with human prostate cancer cell lines, revealed that the IL1β locus is hypermethylated in DU-145 cells in comparison with LNCaP and PC3-ML cells (48). Here, we analyzed DNA methylation levels at the IL1β locus in DU-145 and PC3-ML cells by interrogating the NCI-60 DNA methylome data (GSE49143) and confirmed that the IL1β locus in DU-145 cells is hypermethylated (Fig. 4B).

To delve more into the idea that DNA methylation could repress IL1β transcription when the AR is inactive, we sought to determine whether loss of DNA methylation in DU-145 cells would upregulate IL1β. To this end, we treated these cells with 5-azacytidine, a DNA methyltransferase inhibitor, and indeed found that this significantly increased IL1β transcript expression (Fig. 4C). To validate this *in vitro* observation in the clinical realm, we interrogated the subset of patients with mCRPC that lacked an active AR-signaling axis and yet failed to express IL1β. Results from WGBS conducted on this subset of patients revealed 346 CpG sites spanning the IL1β promoter and gene body on chromosome 2, of which 34 showed significant differences in methylation (Fig. 4D; Supplementary Table S2) when comparing patients with similarly low AR activity but having high versus low expression of IL1β. Interestingly, nine of these 34 CpG sites were detected also by the probes used for DU-145 cells in the GSE49143 methylome data analysis. Notably, only the CpG site at position chr:112830099—detected by probe cg14117934—showed differential methylation between high and low IL1β patients, but the same site was not differentially methylated between PC3-ML and DU-145 cells.

This discrepancy, which is to be expected considering the inevitable differences between established cell lines and human samples, suggests that methylation of multiple CpG sites on the IL1β promoter and gene body modulates the transcription of this cytokine in prostate cancer cells.

## Discussion

Approximately 30% of patients with prostate cancer treated by local modalities develop biochemical recurrence (49), which is announced by an increase in serum of PSA and is commonly treated with ADT alone or combined with ARIs. These strategies temporarily ameliorate or decelerate clinical progression, but most patients eventually transition to a CRPC stage and succumb to metastatic disease (50). Although patients commonly respond to AR-targeted approaches by decreasing serum PSA, it is also common to detect low PSA in the presence of high metastatic burden (51). Thus, disseminated cancer cells with an inactive or mitigated AR-signaling axis can still colonize and grow in target organs. Furthermore, the percentage of patients with mCRPC with a substantial fractions of cancer cells lacking AR has more than tripled over the last decade, due to therapy-induced lineage plasticity (5, 52). We and others (4, 53) have shown that most of these cells do not express neuroendocrine markers—previously always associated with an AR-negative status—and belong to AR-low (ARLPC) or double-negative (DNPC) phenotypes, as described previously (6). Therefore, patients with prostate cancer receiving ADT/ARIs harbor a spectrum of tumor cells, including those with AR expression but a temporarily or permanently inactive AR-signaling axis and those lacking AR altogether (52, 54). This shifting clinical scenario requires novel therapeutic strategies tailored to targeting proliferative and survival pathways that emerge from the suppression of AR signaling (55).

Our study aimed to address these pressing issues and investigate the incidence and mechanistic foundation for how AR signaling regulates IL1β expression in prostate cancer. Our previous work on bone metastasis specimens from 10 patients with prostate cancer showed that AR_POS_ cells harvested by laser capture microdissection and analyzed by RT-PCR completely lacked IL1β transcript (4). Here we interrogated whole-transcriptome RNA-seq data from 100 fresh-frozen biopsies of metastases from patients with mCRPC treated with ADT and/or ARIs and found that lack or reduced transcription of AR-dependent genes is associated with expression of IL1β, a cytokine that is consistently repressed in cancer cells with an active AR-signaling axis. These new findings indicate that the current prostate cancer standard of care will upregulate IL1β in patients presenting with secondary lesions with an exclusive or predominant AR_POS_ status similarly to patients with metastatic tumors harboring mostly or entirely AR_NEG_ tumor cells.

These findings are of high clinical relevance, because IL1β promotes disease progression across multiple cancer types. For instance, IL1β expression by tumors cells can support bone colonization in a humanized mouse model of breast cancer, and treatment with either anakinra or canakinumab, both inhibitors of IL1β signaling, significantly reduce the development of experimental bone metastases (21). In colon cancer, IL1β supports disease progression by inducing epithelial–mesenchymal transition and promotes the emergence of a cancer stem cell phenotype (56). In preclinical models of pancreatic cancer, tumor cells express IL1β to establish an immunosuppressive tumor microenvironment that fosters tumor progression (57).

Our group was the first to propose a prometastatic role for IL1β in prostate cancer based on preclinical studies demonstrating that IL1β expression by PC3-ML cells permits the growth of skeletal disseminated tumors in mice, because short hairpin RNA-mediated knockdown of this cytokine significantly impairs their ability to metastasize (14). Consistently, while DU-145 cells lack IL1β and are nonmetastatic when grafted in the systemic blood circulation of mice, their exogenous expression of IL1β allows DU-145 cells to generate metastatic lesions. Finally, we showed that systemic treatment with Anakinra significantly impairs the ability of PC3-ML cells to grow as disseminated bone tumors (4).

The correlative findings from our RNA-seq analysis of patient data were corroborated by *in vitro* studies. We cultured LNCaP cells, which are hormone sensitive and AR_POS_, in conditions devoid of androgens and observed a time-dependent increase in IL1β expression that never plateaued. In addition, enzalutamide treatment provided results similar to androgen deprivation. Finally, reexpression of AR in PC3-ML cells and DHT treatment completely repressed both IL1β expression at the transcriptional level and secretion of IL1β protein. A similar outcome was observed in animal studies in which LNCaP cells harvested from osseous tumors of mice treated with enzalutamide robustly upregulated IL1β.

We revealed the mechanistic basis for our findings by showing that AR represses IL1β transcription by interacting with an ARE half-site located on the gene promoter, thus explaining why either lack of expression or functional inactivation of this receptor derepresses the cytokine. Because we also show that histone acetylation—specifically at the H3K27Ac mark—promotes IL1β transcription, we posited that AR repression of IL1β requires the recruitment of one or more HDACs to the chromatin, as previously shown for other target genes (58). This idea was corroborated by our studies using the two BET inhibitors JQ1 and PLX51107. Currently, there is significant interest in using BET inhibitors to treat aggressive prostate cancer (59, 60) and a recent study reported high antitumor activity of BET inhibitors on cellular models of CRPC (61).

In light of the existing literature and the novel findings reported here, and because the vast majority of patients with mCRPC inexorably progress to an incurable stage despite AR-targeted treatments, it would be fitting to pursue blocking IL1β signaling in the clinic. This approach seems especially indicated for patients with prostate cancer harboring significant fractions of AR_NEG_ tumor cells—either at earlier stages or upon developing these cellular variants when starting ADT/ARIs. Prostate cancer cells lacking AR are inherently refractory to AR-targeted therapies and can express IL1β at any stage of clinical progression. In fact, the additional ablation of AR_NEG_ tumor cells likely explains the outcome of clinical trials in which AR-agnostic drugs such as docetaxel, administered either prior to (CHAARTED; ref. 62) or in combination with ADT (STAMPEDE; ref. 63) in early metastatic prostate cancer resulted in longer overall survival.

Most notably, our study demonstrates that even patients with predominantly AR_POS_ metastases that respond to ADT and ARIs with a rapid decline in PSA—will have their disseminated tumors effectively converted into an AR-inactive functional state. The subset of patients that lack methylation of the IL1β locus will greatly upregulate this cytokine and could be preemptively identified by liquid biopsies for circulating tumor cells (CTC) or circulating DNA (ctDNA) combined with methodologies currently being implemented for other tumors and biomarkers (64–67). These patients would be ideally apt to benefit from therapeutics that directly affect IL1β signaling like the mAb gevokizumab (68), which is currently being tested for metastatic colorectal, gastroesophageal, and renal cancers in a phase Ib study (NCT03798626) and for patients with colon cancer in a phase II/III study (NCT05178576) upon previous screening by ctDNA.

In conclusion, this study presents strong evidence supporting the adoption of anti-IL1β treatment strategies for patients with prostate cancer to be combined with AR-targeted therapies, which could address both AR_NEG_ and AR-inactivated tumor phenotypes that emerge due to current standard of care, thereby increasing the likelihood of curative outcomes.

## Supplementary Material

Figure S1 C4-2B cells exposed to Enzalutamide show a dose-dependent(a) and time- dependent increase in IL-1β expression (b). Removal of enzalutamide after a 15-day treatment resulted in a return of IL-1β expression to control (undetectable) levels (c). Same results were obtained with 15-day hormone-deprivation followed by DHT re-addition (d).

Figure S2 Expression of CMV-AR and TRE-AR constructs in PC3-ML cells, confirmed at the transcript level for both (a) and at the protein level for the TRE-AR following addition of doxycycline to the culture medium (b).

Figure S3 PC3-ML cells transduced with an inducible AR-overexpression construct showed strong mitigation of IL-1β expression (a) and secretion (b), as compared to both wild type, non-transduced cells and transduced cells without doxycycline exposure.

Figure S4 PC3-ML cells show no difference in luciferase activity between the wild-type promoter and the AREΔ IL-1β promoter, in which the ARE half-site located at -576 was removed.

Table TS1 Primer sequences used for ChIP assay

Table TS2 Whole genome bisulfite sequencing conducted on a subset of patients with low AR activity and low IL-1β expression revealed 346 CpG sites spanning the IL-1β promoter and gene body on chromosome 2, of which 34 showed significant differences in methylation when comparing patients with similarly low AR activity but having high vs. low expression of IL-1β.
