# Supplementary material for: IL1β Expression Driven by Androgen Receptor Absence or Inactivation Promotes Prostate Cancer Bone Metastasis
Source: Cancer Res Commun. 2022 Dec 2;2(12):1545–57. doi: 10.1158/2767-9764.CRC-22-0262 (PMC9770512; doi:10.1158/2767-9764.CRC-22-0262)
Supplement: Figure S1 — C4-2B cells exposed to Enzalutamide show a dose-dependent(a) and time- dependent increase in IL-1β expression (b). Removal of enzalutamide after a 15-day treatment resulted in a return of IL-1β expression to control (undetectable) levels (c). Same results were obtained with 15-day hormone-deprivation followed by DHT re-addition (d). [file crc-22-0262-s01.pptx]

## Slide 1
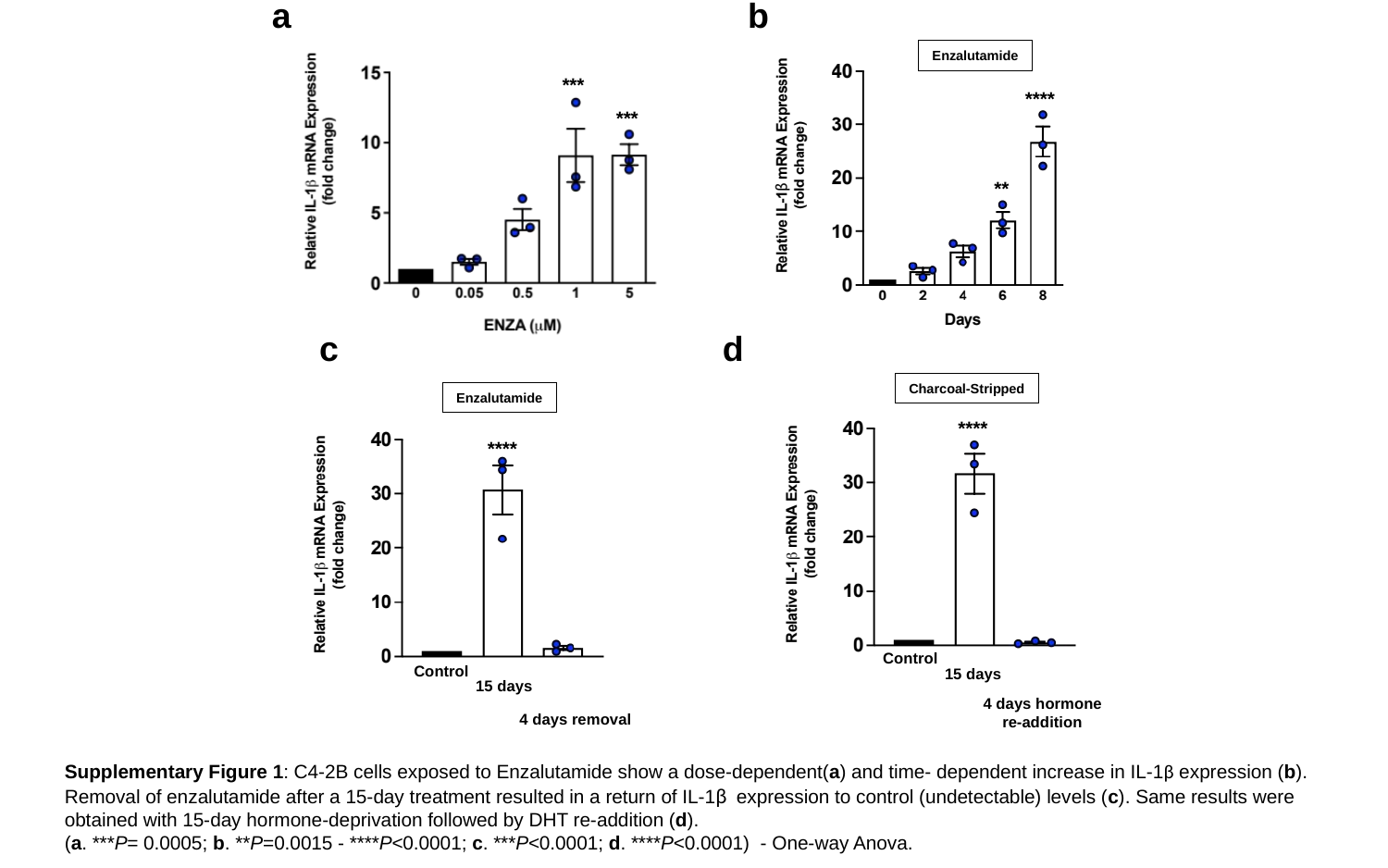

a
b
Enzalutamide
***
****
***
**
c
d
Charcoal-Stripped
Enzalutamide
****
****
Control
Control
15 days
15 days
4 days hormone
re-addition
4 days removal
Supplementary Figure 1: C4-2B cells exposed to Enzalutamide show a dose-dependent(a) and time- dependent increase in IL-1β expression (b).
Removal of enzalutamide after a 15-day treatment resulted in a return of IL-1β expression to control (undetectable) levels (c). Same results were
obtained with 15-day hormone-deprivation followed by DHT re-addition (d).
(a. ***P= 0.0005; b. **P=0.0015 - ****P<0.0001; c. ***P<0.0001; d. ****P<0.0001) - One-way Anova.
