# Supplementary material for: IL1β Expression Driven by Androgen Receptor Absence or Inactivation Promotes Prostate Cancer Bone Metastasis
Source: Cancer Res Commun. 2022 Dec 2;2(12):1545–57. doi: 10.1158/2767-9764.CRC-22-0262 (PMC9770512; doi:10.1158/2767-9764.CRC-22-0262)
Supplement: Figure S2 — Expression of CMV-AR and TRE-AR constructs in PC3-ML cells, confirmed at the transcript level for both (a) and at the protein level for the TRE-AR following addition of doxycycline to the culture medium (b). [file crc-22-0262-s02.pptx]

## Slide 1
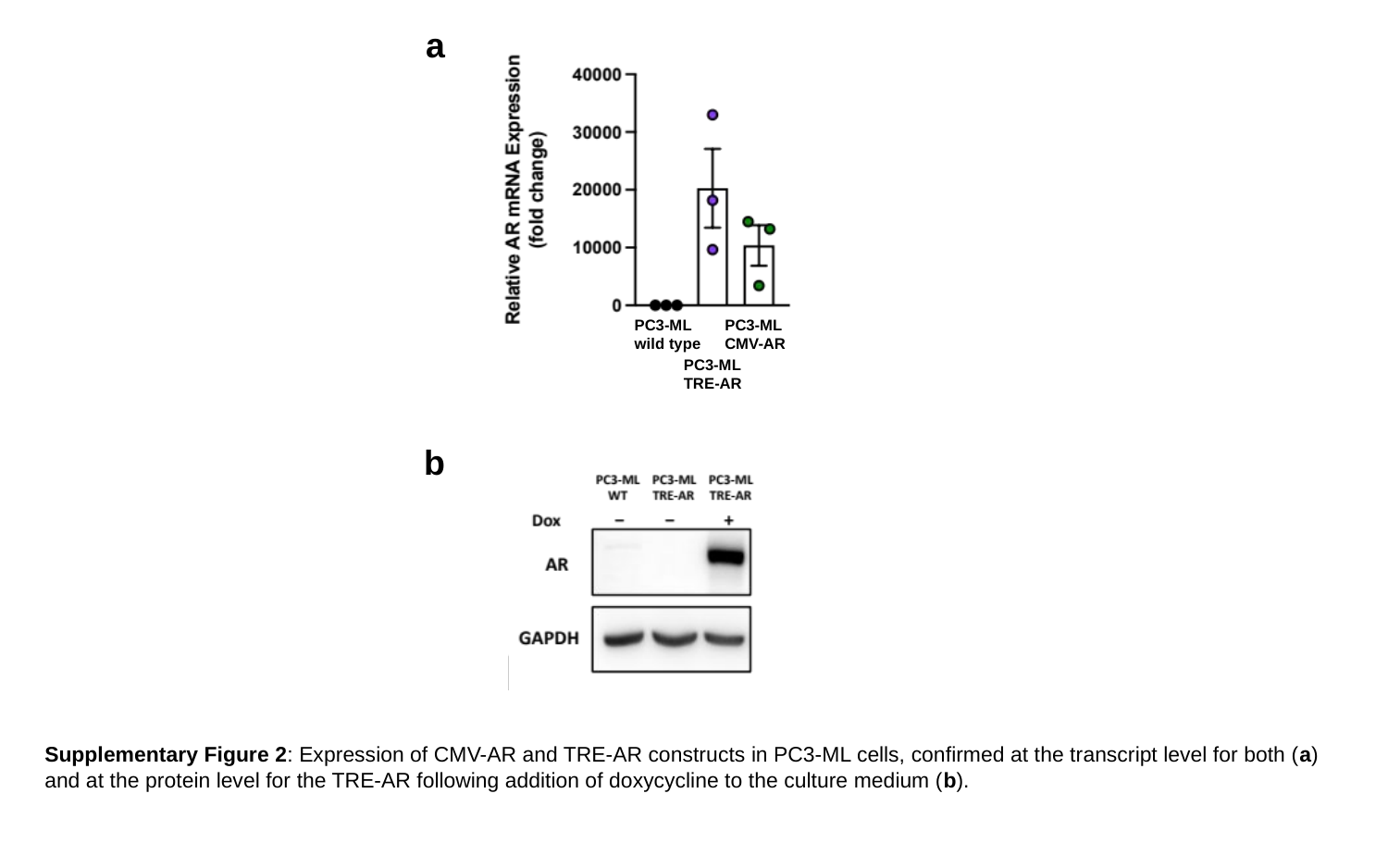

a
PC3-ML
wild type
PC3-ML
CMV-AR
PC3-ML
TRE-AR
b
Supplementary Figure 2: Expression of CMV-AR and TRE-AR constructs in PC3-ML cells, confirmed at the transcript level for both (a)
and at the protein level for the TRE-AR following addition of doxycycline to the culture medium (b).
