# Supplementary material for: IL1β Expression Driven by Androgen Receptor Absence or Inactivation Promotes Prostate Cancer Bone Metastasis
Source: Cancer Res Commun. 2022 Dec 2;2(12):1545–57. doi: 10.1158/2767-9764.CRC-22-0262 (PMC9770512; doi:10.1158/2767-9764.CRC-22-0262)
Supplement: Figure S3 — PC3-ML cells transduced with an inducible AR-overexpression construct showed strong mitigation of IL-1β expression (a) and secretion (b), as compared to both wild type, non-transduced cells and transduced cells without doxycycline exposure. [file crc-22-0262-s03.pptx]

## Slide 1
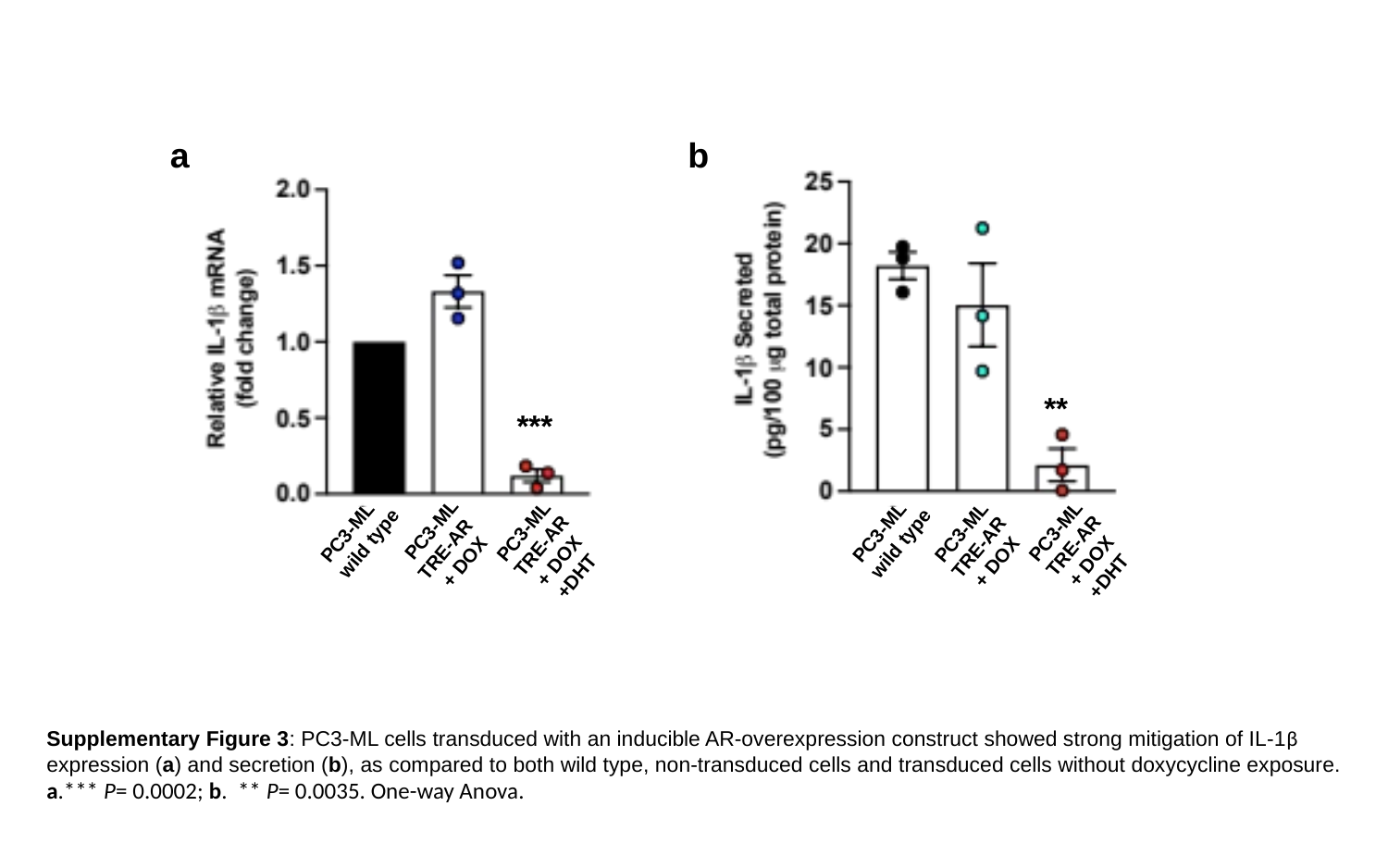

a
b
**
***
PC3-ML
TRE-AR
+ DOX
+DHT
PC3-ML
TRE-AR
+ DOX
+DHT
PC3-ML
TRE-AR
+ DOX
PC3-ML
TRE-AR
+ DOX
PC3-ML
wild type
PC3-ML
wild type
Supplementary Figure 3: PC3-ML cells transduced with an inducible AR-overexpression construct showed strong mitigation of IL-1β
expression (a) and secretion (b), as compared to both wild type, non-transduced cells and transduced cells without doxycycline exposure.
a.*** P= 0.0002; b. ** P= 0.0035. One-way Anova.
