# Supplementary material for: IL1β Expression Driven by Androgen Receptor Absence or Inactivation Promotes Prostate Cancer Bone Metastasis
Source: Cancer Res Commun. 2022 Dec 2;2(12):1545–57. doi: 10.1158/2767-9764.CRC-22-0262 (PMC9770512; doi:10.1158/2767-9764.CRC-22-0262)
Supplement: Figure S4 — PC3-ML cells show no difference in luciferase activity between the wild-type promoter and the AREΔ IL-1β promoter, in which the ARE half-site located at -576 was removed. [file crc-22-0262-s04.pptx]

## Slide 1
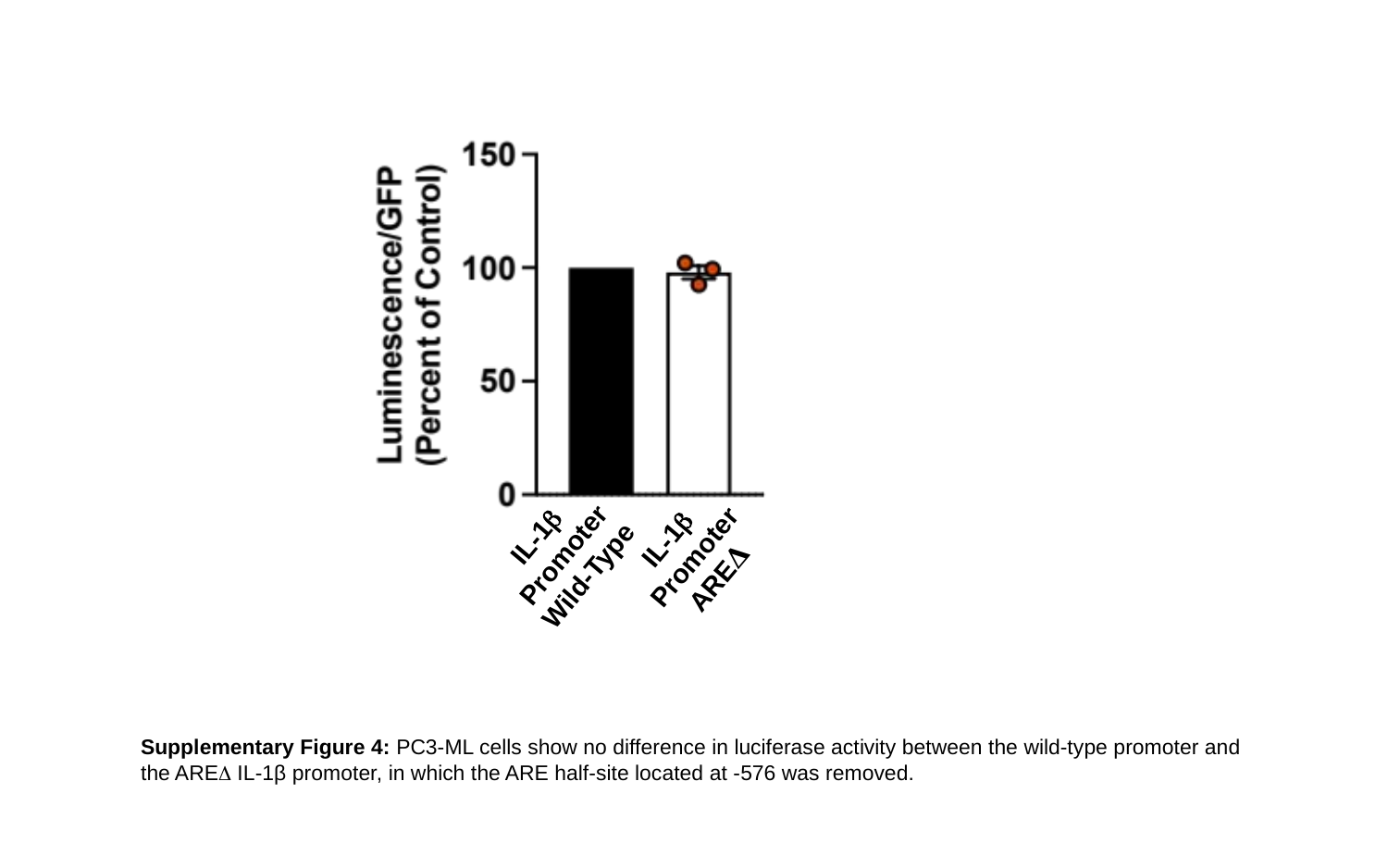

IL-1b
Promoter
Wild-Type
IL-1b
Promoter
ARE𝚫
Supplementary Figure 4: PC3-ML cells show no difference in luciferase activity between the wild-type promoter and
the AREΔ IL-1β promoter, in which the ARE half-site located at -576 was removed.
