# Supplementary material for: IL1β Expression Driven by Androgen Receptor Absence or Inactivation Promotes Prostate Cancer Bone Metastasis
Source: Cancer Res Commun. 2022 Dec 2;2(12):1545–57. doi: 10.1158/2767-9764.CRC-22-0262 (PMC9770512; doi:10.1158/2767-9764.CRC-22-0262)
Supplement: Table TS1 — Primer sequences used for ChIP assay [file crc-22-0262-s05.pdf]

TARGET	PRIMER SEQUENCE
PABPC1 F	GGCGCGGGGTATAAGTAGAG
PABPC1 R	CTCCGCACTCTCAGCACTAA
KLK3 F	CCTCAATCTTATACTGGGACAACT
KLK3 R	GTAGGTCTGTTTTCAATCCAAGA
IL1β -619 to -487 F	TTGCCCCAACTCCGTCAG
IL1β -619 to -487 R	CCTTGGGTGCTGTTCTCTG
IL1β -506 to -250 F	GCAGAGAACAGCACCCAAG
IL1β -506 to -250 R	CAATCGTTGTGCAGTTGATGT

Supplementary Table 1 - Primer sequences used for ChIP assay
