# Supplementary material for: IL1β Expression Driven by Androgen Receptor Absence or Inactivation Promotes Prostate Cancer Bone Metastasis
Source: Cancer Res Commun. 2022 Dec 2;2(12):1545–57. doi: 10.1158/2767-9764.CRC-22-0262 (PMC9770512; doi:10.1158/2767-9764.CRC-22-0262)
Supplement: Table TS2 — Whole genome bisulfite sequencing conducted on a subset of patients with low AR activity and low IL-1β expression revealed 346 CpG sites spanning the IL-1β promoter and gene body on chromosome 2, of which 34 showed significant differences in methylation when comparing patients with similarly low AR activity but having high vs. low expression of IL-1β. [file crc-22-0262-s06.pdf]

Supplementary Table 2

CpG position	Wilcoxon P-value	Mean Methylation High IL-1β	Mean Methylation Low IL-1β	DU-145 cells
112819763	0.837970272	0.918612076	0.92153282	
112819764	0.916404977	0.913731663	0.909236749	
112819810	0.116874188	0.914061527	0.965443122	
112819811	0.819598738	0.957831064	0.966391479	
112819850	0.237353549	0.891597398	0.912256144	
112819851	0.041249699	0.88663473	0.950548073	
112819874	0.822470983	0.953755384	0.949256277	
112819875	0.258190291	0.941956195	0.967397606	
112819927	0.128393117	0.770407843	0.826896043	
112819928	0.332627942	0.834714408	0.846016385	
112819947	0.837062603	0.967781863	0.961315394	
112819948	0.093557581	0.967710129	0.944154864	
112819974	0.416817117	0.969636874	0.963998538	
112819975	0.666689317	0.963848502	0.96978022	
112821193	0.425241384	0.741421376	0.819301885	
112821194	0.557381667	0.726990565	0.788441157	
112821246	0.71707744	0.759434706	0.754999216	
112821247	0.055090925	0.731976295	0.827818702	
112821252	0.397746134	0.669219856	0.712832416	
112821253	0.23250709	0.672810323	0.735993125	
112821471	0.499719454	0.840767951	0.873835763	
112821472	0.283396659	0.835256318	0.873191651	
112821516	0.087119894	0.752982764	0.835956697	
112821517	0.490053481	0.776200214	0.810705081	
112821766	0.208292468	0.83963382	0.890520275	
112821767	0.986170213	0.870323841	0.850562975	
112821839	0.027930942	0.621323173	0.777306764	
112821840	0.13305697	0.647713707	0.784004962	
112821857	0.09751064	0.718347079	0.844791876	
112821858	0.220343351	0.705696567	0.812386517	
112821963	0.189612618	0.654555305	0.762036855	
112821964	0.568883962	0.687531362	0.746806854	
112822247	0.161881184	0.817865755	0.882601207	
112822248	0.48958848	0.826856013	0.877187436	
112822368	0.663246327	0.785650624	0.85393886	
112822369	1	0.822263759	0.840003986	
112822481	0.039714126	0.80332145	0.92726822	
112822482	0.043400924	0.822616969	0.896761228	
112822516	0.056259615	0.722982573	0.837373502	
112822517	0.863000486	0.828954625	0.808057423	
112823410	0.843900087	0.902099116	0.918393258	
112823411	0.362058944	0.915881774	0.932494357	
112823416	0.769803156	0.940787338	0.95526165	

Supplementary Table 2

112823417	0.82486539	0.949590979	0.925477378	
112823483	1	0.894530072	0.857458709	
112823484	0.567905038	0.870591476	0.879704572	
112823631	0.807279824	0.96031746	0.970408163	
112823632	0.866378043	0.927677618	0.942034726	
112823639	0.286023221	0.937604063	0.983928571	
112823640	0.533228468	0.986805556	0.969640853	
112823647	0.347884588	0.951923077	0.975213675	
112823648	0.118500763	0.92478836	0.936544397	
112823656	0.454387213	0.951502268	0.971940559	
112823657	0.772610558	0.964508177	0.955953768	
112823663	0.291289251	0.988888889	0.962894249	
112823664	0.93952704	0.980606061	0.979819563	
112823676	0.892469908	0.943095238	0.949158307	
112823677	0.538366304	0.947111315	0.953005074	
112823684	0.413866595	0.842573696	0.877876984	
112823685	0.713675148	0.842093462	0.862338202	
112823701	0.020124995	0.869756236	0.957118056	
112823702	0.828687876	0.951587302	0.967136644	
112823708	0.648176175	0.81037296	0.773227513	
112823709	0.769820604	0.869314759	0.831489321	
112823716	0.817698184	0.962554113	0.873214286	
112823717	0.287593214	0.927550505	0.85048787	
112823740	0.761934637	0.912777778	0.905924036	
112823741	0.867008527	0.893596681	0.892735941	
112823760	0.508841299	0.980867347	0.991666667	
112823761	0.001648399	0.946454934	1	
112823764	0.497437062	0.969387755	0.9875	
112823765	0.173675275	0.949033605	0.971122995	
112823770	0.962837947	0.993506494	0.994117647	
112823771	0.35772862	0.953805426	0.968322095	
112823772	0.28695789	0.96122449	0.983660131	
112823773	0.171940626	0.966593823	0.980882353	
112823776	0.216094947	0.959368409	0.979055258	
112823777	0.129612634	0.964457071	0.986859546	
112823805	0.841302535	0.948994709	0.954166667	
112823806	0.406421773	0.9276934	0.949296645	
112823836	0.781408019	0.949163832	0.943859227	
112823837	0.651922817	0.912423774	0.909088135	
112823861	0.460898681	0.939617605	0.972811448	
112823862	1	0.950825349	0.945348378	
112823866	0.763072082	0.928508899	0.944047002	
112823867	0.185612809	0.92077743	0.947999564	
112823894	0.15105351	0.930980392	0.959615385	
112823895	0.80361317	0.931933594	0.932138767	
112823904	0.635741378	0.955441694	0.961866506	

Supplementary Table 2

112823905	0.846662615	0.95782	0.966553014	
112823906	0.11783143	0.953334288	0.976670298	
112823907	0.033681418	0.953426182	0.982998768	
112823910	0.145347337	0.903196177	0.952073906	
112823911	0.465866333	0.912982171	0.959749672	
112823950	0.135909335	0.918757407	0.961071608	
112823951	0.81744178	0.956344642	0.942507796	
112824132	0.246366562	0.788659839	0.869464476	
112824133	0.580710019	0.834339694	0.853219181	
112824147	0.600947546	0.915054365	0.909646665	
112824148	0.875030491	0.913560573	0.921155726	
112824276	0.479162768	0.862928032	0.831926628	
112824277	0.342037266	0.864376955	0.865574675	
112824290	0.69619618	0.88557675	0.860743533	
112824291	0.742944504	0.865696583	0.829250906	
112824421	1	0.664082195	0.651727363	
112824422	0.276954826	0.691656731	0.624334538	
112824453	0.641235346	0.70590134	0.690895309	
112824454	0.742982423	0.749108457	0.736149992	
112824564	0.657051024	0.172406046	0.143689983	
112824565	0.072720357	0.224553967	0.150334062	
112825121	0.094433024	0.838971116	0.917575926	
112825122	0.336498554	0.890409821	0.927693103	
112825227	0.089722149	0.933988858	0.972959857	
112825228	0.957207747	0.945365161	0.950107515	
112825260	0.171534472	0.926077272	0.957860049	
112825261	0.086965849	0.930376295	0.958856155	
112825495	0.023162386	0.79157523	0.883818783	
112825496	0.120252144	0.784240256	0.836513707	
112825681	0.185491971	0.87923847	0.927398511	
112825682	0.488018163	0.906952322	0.915557287	
112825692	0.397059522	0.845179991	0.857054388	
112825693	0.378730852	0.853602597	0.882190884	
112825852	0.768690832	0.872102418	0.882185806	
112825853	0.227124478	0.821741655	0.863427971	
112825984	0.377319783	0.933329131	0.968549899	
112825985	0.671909616	0.954359025	0.95274454	
112826168	0.7686563	0.875364029	0.850525884	
112826169	0.835597829	0.865010494	0.837702996	
112826198	0.74141384	0.924187336	0.899727667	
112826199	0.54418234	0.897173097	0.8617129	
112826264	0.971658962	0.949356954	0.926216153	
112826265	0.322126111	0.924471745	0.91042456	
112826550	0.06270303	0.912495768	0.939733341	
112826551	0.063141907	0.909434491	0.92898877	
112826671	0.495001649	0.916569736	0.958852466	

Supplementary Table 2

112826672	0.022828992	0.915010295	0.959014322	
112826854	0.484354818	0.916699447	0.934862897	
112826855	0.560361044	0.937234578	0.934523443	
112826859	0.460552206	0.96013604	0.948460892	
112826860	0.735833093	0.953371943	0.958583815	
112827167	0.070984296	0.925106838	0.948940003	
112827168	0.123785428	0.924734479	0.953948526	
112827180	0.567288356	0.823339347	0.693300654	
112827181	0.972393488	0.544810878	0.548836273	
112827204	0.884995096	0.949887737	0.950526179	
112827205	0.704157141	0.958297274	0.950546684	
112827467	0.091838118	0.942267769	0.931642642	
112827468	0.110160183	0.920891977	0.94608858	
112827699	0.874433634	0.879700221	0.862321161	
112827700	0.638972218	0.90431468	0.900310546	
112827756	0.903792158	0.759835222	0.738764317	
112827757	0.29995745	0.794460129	0.73715296	
112827856	0.876368767	0.818441934	0.806373751	
112827857	0.278843167	0.770674719	0.798569988	
112827937	0.230102947	0.939291338	0.888580383	
112827938	0.393806956	0.865424679	0.905976354	
112828043	0.478629243	0.815097687	0.844204341	
112828044	0.876038232	0.871305964	0.864861708	
112828135	0.795763391	0.69020997	0.657131396	
112828136	0.351512429	0.73255973	0.644877876	
112828470	0.057050867	0.72815458	0.846824538	
112828471	0.204895809	0.761523598	0.83841855	
112828472	0.308343934	0.802507539	0.861808398	
112828473	0.377524472	0.82036193	0.873336466	
112828498	0.568943078	0.661036729	0.616327027	
112828499	0.417251433	0.638156487	0.575268038	
112828804	0.116379467	0.92817051	0.967254812	
112828805	0.574886419	0.944824693	0.967980582	
112828831	0.041371145	0.900906515	0.963415762	
112828832	0.124772557	0.901443283	0.948090012	
112828993	0.018982344	0.912498873	0.972242989	
112828994	0.587692912	0.918332053	0.955793924	
112829181	0.070630461	0.922072414	0.952356113	
112829182	0.531241905	0.937271998	0.926976244	
112829223	0.85992451	0.879262975	0.902413404	
112829224	0.476424137	0.842745758	0.89909099	
112829291	0.568972627	0.501105519	0.54954675	
112829292	0.437417766	0.558412254	0.608169164	
112829445	0.426831966	0.835494387	0.862599206	
112829446	0.957513368	0.883555711	0.883567123	
112829610	0.795732856	0.715432135	0.636471787	

Supplementary Table 2

112829611	0.308306912	0.732284236	0.652416704	
112830023	0.045401647	0.88275689	0.941099777	
112830024	0.031693404	0.884574195	0.949182299	
112830047	0.348288014	0.869936458	0.916046247	
112830048	0.039356546	0.885973962	0.949588544	
112830082	0.504790421	0.898841373	0.914723269	
112830083	0.448511513	0.896781517	0.91833513	
112830098	0.313597291	0.863929438	0.918662463	
112830099	0.014854791	0.854179314	0.954079589	cg14117394 in DU-145 cells
112830274	0.006748676	0.89140995	0.955183062	
112830275	0.275056603	0.916248929	0.96271954	
112830405	0.051032423	0.873434185	0.941164929	
112830406	0.534248765	0.890750169	0.917379465	
112830429	0.069968047	0.893008895	0.95171213	
112830430	0.237280828	0.865524484	0.902450171	
112830530	0.588168407	0.907920072	0.938882476	
112830531	0.068392182	0.880781892	0.942249626	
112830580	0.834453875	0.918255018	0.917470905	
112830581	0.210348101	0.913841612	0.934473705	
112830666	0.170021374	0.851958193	0.893664296	
112830667	0.385043099	0.875209634	0.910658012	
112830725	0.545047202	0.807250782	0.774122294	
112830726	0.808474233	0.616146139	0.564825634	
112831088	0.111734769	0.911781981	0.927566428	
112831089	0.216032522	0.923044294	0.918845316	
112831131	0.715259747	0.946308415	0.906807558	
112831132	0.258312188	0.925986809	0.926765778	
112831216	0.042152258	0.662465737	0.825547027	
112831217	0.142237144	0.555554515	0.682250786	
112831327	0.767156647	0.887695601	0.895542973	
112831328	0.019084368	0.846454337	0.93030827	
112831662	0.479302223	0.654360148	0.736946417	
112831663	0.095831319	0.660359559	0.791622787	
112831756	0.829281407	0.650942507	0.642153121	
112831757	0.717303139	0.548137233	0.525356434	
112831977	0.50100026	0.591949388	0.651882141	
112831978	0.756013904	0.604880143	0.620273652	
112832107	0.333406671	0.663104843	0.753222259	
112832108	0.534279606	0.687608495	0.722873513	
112832146	0.396914681	0.704821935	0.817432588	
112832147	0.030842754	0.661023604	0.819511584	
112832165	0.248026436	0.674928775	0.790875628	
112832166	0.051674745	0.615314749	0.772236111	
112832173	0.282969009	0.754894735	0.862440858	
112832174	0.003514377	0.669924927	0.855704675	
112832198	0.198465362	0.761481481	0.89506734	

Supplementary Table 2

112832199	0.031572632	0.678994386	0.847053526	
112832241	0.336103522	0.812962963	0.738095238	
112832242	0.122176206	0.704554189	0.847109139	
112832291	0.167254862	0.708641975	0.867861504	
112832292	0.14138178	0.707873461	0.844953288	
112832444	0.128217519	0.741129802	0.84246973	
112832445	0.233080861	0.763887884	0.873618282	
112832748	0.278816915	0.921733736	0.955776615	
112832749	0.035064557	0.928022168	0.981479686	
112832753	0.149917368	0.920801334	0.953155726	
112832754	0.187590331	0.936925703	0.968958819	
112832769	0.459030727	0.95348233	0.974962026	
112832770	0.462438163	0.953693443	0.971301002	
112832782	0.019910675	0.906799818	0.966330809	
112832783	0.038468393	0.918374138	0.952745188	
112832797	0.627427956	0.87678761	0.900150218	
112832798	1	0.914639772	0.894006655	
112832812	0.354251048	0.774974613	0.830408974	
112832813	0.970175677	0.823369829	0.818111934	
112832878	0.078787178	0.905904399	0.957712326	
112832879	0.389703911	0.918456441	0.967134678	
112832885	0.17382897	0.947234825	0.972731428	
112832886	0.4796263	0.97223136	0.976617344	
112832890	0.649036423	0.962980296	0.88746171	
112832891	0.765068809	0.736049285	0.653570707	
112832929	0.193893602	0.942767543	0.966575547	
112832930	0.427719541	0.952004892	0.94101843	
112833050	0.063123924	0.874310241	0.915313823	
112833051	0.170946227	0.886502512	0.896563054	
112833112	0.481580219	0.955116582	0.956635953	
112833113	0.678112716	0.93488957	0.939020412	
112833259	0.506399722	0.921168302	0.935542455	
112833260	0.971205446	0.938954551	0.935825439	
112833480	0.052757094	0.94002187	0.979193035	
112833481	0.312356028	0.914785028	0.964168045	
112833482	0.076982255	0.948109339	0.989417989	
112833483	0.884423139	0.948603932	0.970921517	
112833515	0.015788193	0.875712344	0.921516111	
112833516	0.985603989	0.894899111	0.924510486	
112833523	0.162055271	0.872529949	0.908843673	
112833524	0.27873912	0.847845965	0.901153433	
112833536	0.834555611	0.955040862	0.944807928	
112833537	0.686186655	0.933399321	0.949445953	
112833579	0.792841141	0.969079969	0.976435374	
112833580	0.245496839	0.946663233	0.962400487	
112833588	0.985086571	0.951485873	0.944682194	

Supplementary Table 2

112833589	0.031256001	0.884245977	0.920954586	
112833685	0.308306912	0.815164013	0.822079167	
112833686	0.690451401	0.819229739	0.815618336	
112833719	0.793859613	0.915850489	0.913473295	
112833720	0.833310629	0.92152775	0.9303735	
112833759	0.034479922	0.838332174	0.913031644	
112833760	0.138643068	0.853790682	0.899177869	
112833813	0.013323792	0.90445402	0.963999867	
112833814	0.663221718	0.906174914	0.922388601	
112833907	0.139017397	0.872104525	0.936969882	
112833908	0.021975313	0.878789549	0.948677277	
112834457	0.742963465	0.479049684	0.431553318	
112834458	0.388186062	0.498304049	0.422320921	
112835037	0.043960343	0.859712506	0.924298914	
112835038	0.156872176	0.86380288	0.940746602	
112835370	0.087313622	0.727064471	0.833434523	
112835371	0.094171361	0.701810811	0.806249829	
112835770	0.137390375	0.85373331	0.916847405	
112835771	0.329449302	0.812065052	0.886176754	
112836170	0.156953742	0.686869067	0.769997735	
112836171	0.22662692	0.692276397	0.793742724	
112836208	0.292491484	0.670693186	0.772258131	
112836209	0.188629452	0.667161581	0.797313725	cg15836722 in DU-145 cells
112836282	0.025640933	0.680624069	0.848018085	
112836283	0.217506847	0.723094627	0.84550666	cg01290568 in DU-145 cells
112836288	0.120053564	0.696412484	0.817406032	
112836289	0.206609117	0.73426934	0.850209984	
112836522	0.501032974	0.415163715	0.4923615	
112836523	0.160485856	0.414772407	0.547159377	
112836766	0.822519534	0.314851846	0.343424292	
112836767	0.835796523	0.358406639	0.381774463	cg20157753 in DU-145 cells
112836798	0.240679239	0.380239564	0.459314502	
112836799	0.903799485	0.463541667	0.453129263	cg07935264 in DU-145 cells
112837034	1	0.327314978	0.344889552	
112837035	0.333628691	0.285407852	0.368624015	cg18773937 in DU-145 cells
112837077	0.195538536	0.463283362	0.559713983	
112837078	0.862990118	0.528416946	0.567490717	cg23149881 in DU-145 cells
112837361	0.768275427	0.870506541	0.879212443	
112837362	0.053209391	0.763527787	0.856307375	
112837386	0.096075233	0.876696719	0.9111777	
112837387	0.005627642	0.820234611	0.935584768	
112837608	0.566893314	0.8937286	0.945246866	
112837609	0.08348635	0.878389826	0.944156335	
112837787	0.020539895	0.848068093	0.916159493	
112837788	0.290454314	0.852933065	0.861724554	
112837811	0.606505391	0.878193619	0.904216296	

Supplementary Table 2

112837812	0.735791922	0.904789576	0.902063529	
112837817	0.848954101	0.817144209	0.817990801	
112837818	0.67796143	0.850509289	0.834685449	
112837834	0.104631083	0.898491994	0.93076151	
112837835	0.439481796	0.935991622	0.927039594	
112837841	0.666017014	0.738344208	0.715757569	
112837842	0.782121892	0.78489641	0.743697619	
112837900	0.026342427	0.903860447	0.955836561	
112837901	0.984245252	0.926759442	0.916000141	
112837905	0.112633949	0.912462741	0.970071314	
112837906	0.832703494	0.931232082	0.940883862	
112837909	0.422311644	0.908552309	0.927435065	
112837910	0.436467368	0.921887119	0.92493181	
112837969	0.843963419	0.898356361	0.905792865	
112837970	0.9017037	0.925282775	0.908997435	
112837994	0.095092437	0.930182764	0.892228902	
112837995	0.940398347	0.933614674	0.931094304	
112837999	0.204580134	0.880477451	0.759793547	
112838000	0.944452253	0.863074658	0.797345066	
112838027	0.316578568	0.852514977	0.774045804	
112838028	0.717036044	0.822772665	0.776037547	
112838037	0.369490532	0.734434996	0.651964427	
112838038	0.087531555	0.783272727	0.645686365	cg02596281 in DU-145 cells
112838233	0.462952694	0.903668059	0.880076494	
112838234	0.819454518	0.915139989	0.8820578	
112838239	0.534182568	0.930236772	0.867341169	
112838240	0.831251191	0.928954232	0.881337553	cg18635064 in DU-145 cells

Supplementary Table 2. Whole genome bisulfite sequencing conducted on a subset of patients with low AR activity and low IL-1β expression revealed 346 CpG sites spanning the IL-1β promoter and gene body on chromosome 2, of which 34 showed significant differences in methylation when comparing patients with similarly low AR activity but having high vs. low expression of IL-1β.
